# AI-assisted multimodal analysis of digital media-enabled intergenerational transmission of regional musical intangible cultural heritage: evidence from blind ballad artists' narrative singing in Jindongnan and the Taihang mountains

**DOI:** 10.3389/fpsyg.2026.1953191

**Published:** 2026-09-29

**Authors:** Qiang Xu

**Affiliations:** School of Chinese Language and Literature, Shanxi University, Taiyuan, China

**Keywords:** blind ballad artists, digital media, explainable artificial intelligence, intergenerational transmission, multimodal fusion, narrative singing, quyi, regional musical intangible cultural heritage

## Abstract

Digital media has expanded the dissemination of regional musical intangible cultural heritage (ICH), but online visibility does not necessarily lead to intergenerational transmission. This study proposes an AI-assisted multimodal framework to examine the digital dissemination and transmission pathways of blind ballad artists' narrative singing traditions in Jindongnan and the Taihang Mountains. The dataset comprises 2,136 online videos, 184,572 interaction records, 412 youth questionnaires, and 36 field interviews. To avoid target leakage, normalized 30-day dissemination effectiveness was predicted using only content-intrinsic features, creator metadata, and 24-h early engagement indicators. The Heritage-aware Explainable Dissemination Framework integrates textual, visual, audio, platform, and field-informed cultural features. Across five creator-level splits, it achieved an MAE of 0.412 ± 0.014, an RMSE of 0.552 ± 0.018, and an *R*^2^ of 0.706 ± 0.020. SHAP analysis identified early engagement, cultural embeddedness, youth-oriented interpretation, inheritor visibility, subtitles, and village scenes as key predictors. PLS-SEM further identified a significant statistical indirect pathway linking digital exposure with transmission intention via emotional resonance, cultural identity, and learning participation. Given the cross-sectional questionnaire design, these relationships are interpreted as associations rather than temporal or causal effects. The findings indicate that stronger transmission-oriented digital engagement is associated with culturally embedded representation, interpretive accessibility, and participatory learning.

## 1 Introduction

Intangible cultural heritage (ICH) refers to the practices, representations, expressions, knowledge, and skills that communities, groups, and individuals recognize as part of their cultural heritage (UNESCO, 2003). Regional musical ICH is especially dependent on oral memory, embodied performance, local language, community participation, and repeated intergenerational learning. Unlike tangible heritage, which can be preserved through material conservation, musical ICH remains alive only when it continues to be performed, interpreted, taught, and re-created in social life. Local narrative singing traditions in northern China are often connected with village rituals, seasonal gatherings, family ceremonies, moral storytelling, and community memory. These traditions are not only artistic expressions but also social mechanisms through which local histories, values, and collective identities are transmitted.

In this study, the term “blind ballad artists” refers to a historically formed group of visually impaired local narrative performers in the study region. The term is used to describe a specific cultural and occupational transmission context rather than to reduce the artists to disability identity. In Jindongnan and the Taihang Mountains, blind ballad artists' narrative singing traditions integrate singing, storytelling, instrumental accompaniment, local dialects, moral narration, and community-based cultural memory. These traditions have long relied on face-to-face performance and local apprenticeship, but their transmission environment is being reshaped by short-video platforms and youth-oriented digital media.

Digital media has expanded the circulation space of regional musical ICH. Short-video platforms, online cultural communities, and social media accounts enable traditional music and folk performance to reach younger audiences beyond local villages. Recent studies have shown that TikTok can promote musical ICH through short videos (Cao, 2025), Bilibili can support youth-oriented dissemination of Cantonese Opera (Cen et al., 2024), and Douyin-based ICH accounts can produce different dissemination effects through specific configurations of platform and content factors (Wang et al., 2025). Other research has indicated that digital interactivity, presence, affective response, immersion, and perceived value are associated with cultural inheritance behavior, digital ICH adoption, and continued use intention (Han et al., 2026; Yi C. et al., 2025; Ye W. et al., 2025; Wang and Men, 2025). These studies suggest that digital media is not merely a technical channel for distributing heritage content but also a socio-technical environment in which cultural attention, affective connection, identity recognition, and learning participation are reorganized.

However, three research gaps remain. First, existing studies often measure digital ICH dissemination through platform metrics but underrepresent the cultural semantics embedded in musical performance, such as dialect, repertoire, inheritor presence, performance scene, narrative clarity, and local community context. Second, AI-based heritage studies have mainly focused on archiving, retrieval, restoration, immersive experience, or content generation rather than dissemination prediction and interpretation (Yan et al., 2025a; Liu et al., 2025; Chen et al., 2024; Buragohain et al., 2024; He and Qin, 2024; Long et al., 2026; Ye X. et al., 2025). Third, online engagement studies rarely examine whether digital exposure is connected with intergenerational transmission intention through field-based evidence. Platform data can reveal how content circulates, but it cannot fully explain whether young audiences develop emotional resonance, cultural identity, learning participation, or sustained transmission intention.

From a media-psychological perspective, this third gap is particularly important. Digital exposure can provide the initial opportunity for information processing, but exposure alone does not specify how heritage content becomes affectively meaningful, identity-relevant, or connected with learning-oriented participation. The Elaboration Likelihood Model, narrative transportation, social identity theory, identity-based motivation, and behavioral-intention theory provide complementary explanations for these stages. Accordingly, the psychological component of the present study is treated as a theory-informed associative model rather than as a causal sequence established by the cross-sectional data.

To address these gaps, this study develops an AI-assisted multimodal analytical framework for examining digital-media-enabled dissemination and intergenerational transmission of regional musical ICH. The empirical focus is blind ballad artists' narrative singing traditions in Jindongnan and the Taihang Mountains. This region provides a meaningful empirical setting because blind narrative performers historically connect musical performance with oral history, moral storytelling, ritual events, and community memory. At the same time, their performance contexts are being reshaped by short-video platforms and youth-oriented digital media. This makes the region suitable for examining how local musical ICH is represented, circulated, and reinterpreted in digital environments.

The contributions of this study are threefold. First, it constructs a heritage-oriented multimodal feature system that integrates platform metadata, early engagement, textual semantics, visual context, audio performance features, and field-informed cultural variables. Second, it develops a leakage-controlled and explainable dissemination prediction framework that uses content and early-stage indicators to predict 30-day dissemination effectiveness while preventing final engagement metrics from entering the input features. Third, it links online dissemination analysis with field-based structural equation modeling to examine theory-informed statistical associations among digital exposure, emotional resonance, cultural identity, learning participation, and transmission intention.

The remainder of this paper is organized as follows. Section 2 reviews related work on digital dissemination of musical ICH, AI-assisted cultural heritage representation, and intergenerational transmission in digital environments. Section 3 presents the research design, data sources, variable construction, feature extraction, modeling framework, and implementation details. Section 4 describes the experimental design, baselines, metrics, ablation tests, and robustness checks. Section 5 reports the empirical results. Section 6 discusses theoretical and practical implications, limitations, and future research directions. Section 7 concludes the paper.

## 2 Related work

### 2.1 Digital dissemination of musical intangible cultural heritage

Digital media has become an important infrastructure for cultural heritage communication. Musical ICH is increasingly disseminated through short videos, livestreaming, digital exhibitions, online communities, and immersive platforms. Cao (Cao, 2025) examined musical ICH promotion under TikTok short videos and showed that digital communication can affect public cultural engagement. Cen et al. (Cen et al., 2024) analyzed Cantonese Opera videos on Bilibili and demonstrated that youth-oriented video communities provide a meaningful platform for performing arts ICH dissemination. Wang et al. (Wang et al., 2025) applied fuzzy-set qualitative comparative analysis to Douyin-based ICH dissemination and found that effective dissemination is driven by configurations of platform characteristics, content traits, and user engagement.

Beyond platform visibility, recent studies have increasingly examined user experience and psychological mechanisms. Han et al. (Han et al., 2026) found that digital interactivity and presence were associated with cultural inheritance behavior through flow experience in the digital dissemination of Morin Khuur. Yi et al. (Yi C. et al., 2025) used Affective Events Theory to explain how digital experience shapes ICH dissemination through affective responses. Ye et al. (Ye W. et al., 2025) integrated the Unified Theory of Acceptance and Use of Technology 2 and immersion theory to analyze digital ICH adoption. Wang and Men (Wang and Men, 2025) examined continued use intention in digital ICH games through the stimulus-organism-response model.

Digital heritage evaluation research also indicates that the effectiveness of ICH dissemination should not be reduced to technical visibility or platform traffic. Yan et al. (Yan et al., 2025b) constructed a digital dissemination evaluation indicator system for traditional techniques and emphasized authenticity, integrity, and transmission value. Liu et al. (Liu et al., 2025) further showed that augmented reality, virtual reality, social media, and AI narratives can shape the acceptance of digital ICH experiences. However, these studies mainly evaluate dissemination through platform-level indicators, digital experience, or adoption intention, and rarely model video-level cultural features embedded in musical ICH.

### 2.2 AI-assisted representation of cultural heritage and regional music

AI has increasingly been applied in cultural heritage preservation, representation, retrieval, and interpretation. Chen et al. (Chen et al., 2024) constructed an AI-based ethnic music dataset and demonstrated the value of computational methods for music heritage documentation and analysis. Buragohain et al. (Buragohain et al., 2024) reviewed metaverse applications for cultural heritage and emphasized opportunities and challenges in immersive digitalization. He and Qin (He and Qin, 2024) used a two-stage structural equation modeling-artificial neural network approach to examine how cultural heritage metaverse experiences are associated with offline experience intention. Long et al. (Long et al., 2026) developed a Chinese named entity recognition dataset for ICH, supporting structured cultural knowledge extraction. Ye et al. (Ye W. et al., 2025) proposed ICH-Qwen, a large language model toward Chinese ICH.

Digital heritage studies have also explored restoration, immersive experience, and active engagement. Basu et al. (Basu et al., 2023) surveyed data-driven computing for digital restoration of cultural heritage. Yu et al. (Yu J. et al., 2025) investigated immersive ICH experiences within cultural heritage sites. Ge (Ge, 2024) studied video-streamed ICH related to Chinese ethnic minorities and its relationship with cross-cultural competence. These studies indicate that AI and immersive technologies can support heritage representation, education, interaction, and preservation.

However, AI-based heritage studies have more often focused on archiving, generation, retrieval, restoration, or interaction than on linking multimodal cultural features with empirical dissemination outcomes. For regional musical ICH, AI representation should not be limited to generic text, image, or audio embeddings. It should be combined with heritage-oriented variables such as cultural embeddedness, inheritor visibility, village scene salience, dialectal expression, and youth-oriented interpretive support.

### 2.3 From online engagement to intergenerational transmission

The essence of ICH lies in living continuity. Digital tools may increase visibility, but intergenerational transmission requires emotional resonance, identity recognition, and participatory learning. The Elaboration Likelihood Model (ELM) provides a useful theoretical lens for understanding how audiences process cultural information (Petty and Cacioppo, 1986). Central-route processing involves deeper attention to content quality, cultural meaning, and narrative explanation, whereas peripheral-route processing relies more on surface cues such as title style, visual attractiveness, platform labels, and creator identity. In the context of digital musical ICH, both routes may operate simultaneously. Young audiences may first be attracted by short-video rhythm, subtitles, visual scenes, or comments, and then develop deeper engagement when the content provides culturally meaningful explanations and authentic performance contexts.

Narrative transportation complements the ELM by emphasizing affective and attentional involvement in mediated stories. Transportation involves attention, imagery, and emotional engagement with a narrative world (Green and Brock, 2000). For narrative-singing heritage, digital exposure may therefore be associated with emotional resonance when viewers attend not only to the musical form but also to performer biographies, local stories, family memories, or village contexts.

Social identity theory further suggests that self-definition can be connected with perceived membership in culturally meaningful groups (Tajfel and Turner, 1979). Emotional engagement with locally meaningful narratives may therefore covary with stronger cultural identification. Identity-based motivation provides a subsequent explanation for why identity relevance may be associated with action readiness: when an identity becomes situationally salient, behaviors that appear congruent with that identity can become more psychologically accessible (Oyserman, 2009).

Existing research has provided important insights into digital ICH adoption and user experience, but online engagement is not equivalent to intergenerational transmission. A video may attract attention without producing sustained learning or participation. Therefore, a field-informed framework is needed to examine how digital exposure is statistically associated with emotional, identity-based, and participatory engagement. The present study responds to this gap by combining AI-assisted multimodal dissemination prediction with structural equation modeling and field interviews.

### 2.4 Media-psychological integration and hypotheses

The psychological model was specified as a theory-informed associative sequence rather than as a causal temporal model. Digital exposure (DE) represents contact with relevant digital heritage content. Within the ELM and narrative-transportation perspectives, repeated or meaningful exposure provides opportunities for both peripheral and central processing and for affective involvement in artist narratives, local memories, and cultural scenes. Accordingly:

- H1. Digital exposure is positively associated with emotional resonance.

Emotional resonance (ER) captures affective connection with performers, stories, rural memories, and local cultural meanings. Social identity theory implies that emotionally meaningful cultural cues can be associated with stronger perceived belonging and cultural self-definition. Accordingly:

- H2. Emotional resonance is positively associated with cultural identity.

Cultural identity (CI) refers to perceived identification with the cultural meaning represented by the heritage tradition. Identity-based motivation proposes that salient identities are associated with action and procedural readiness (Oyserman, 2009). In the present context, identity relevance may therefore be associated with willingness to move from viewing toward learning-oriented engagement. Accordingly:

- H3. Cultural identity is positively associated with learning participation.

Learning participation (LP) represents self-reported engagement with learning-oriented activities, such as seeking explanations, attempting basic singing segments, attending related activities, or communicating about the tradition. Such engagement provides a proximal experiential basis for future participation and is expected to be associated with transmission intention. Accordingly:

- H4. Learning participation is positively associated with transmission intention.

The model also examined three direct associations because digital exposure, emotional resonance, and cultural identity may retain associations with transmission intention that are not fully represented by the sequential path:

- H5. Digital exposure is positively associated with transmission intention.
- H6. Emotional resonance is positively associated with transmission intention.
- H7. Cultural identity is positively associated with transmission intention.

Finally, the theoretical sequence specifies a statistical indirect association across the four intermediate links:

- H8. Digital exposure has a positive statistical indirect association with transmission intention through the sequential pattern of emotional resonance, cultural identity, and learning participation.

Transmission intention is treated as a proximal psychological outcome rather than observed intergenerational transmission. The Theory of Planned Behavior identifies behavioral intention as an important precursor of behavior while recognizing that enactment also depends on behavioral control and enabling conditions (Ajzen, 1991). Empirical work on the intention–behavior gap likewise shows that changes in intention are only partially translated into actual behavior (Webb and Sheeran, 2006). These distinctions are particularly important for heritage transmission, which may require access to inheritors, community learning spaces, repertoire materials, and opportunities for repeated offline participation.

## 3 Materials and methods

### 3.1 Research design and study area

This study consisted of two analytically connected but statistically separate stages. Stage 1 used video-level platform and multimodal data to predict 30-day dissemination effectiveness. Stage 2 used respondent-level questionnaire and interview data to examine the association between digital exposure and transmission intention. The two stages were connected theoretically through digital-media-enabled dissemination and transmission, but they were not merged into a single statistical unit.

The study area included villages and township-level cultural spaces in Jindongnan and the Taihang Mountain region. These areas preserve local narrative singing traditions historically associated with blind ballad artists, ritual occasions, family memory, oral storytelling, and community-based cultural education. Fieldwork was conducted in eight villages and cultural centers. The online component collected videos from Bilibili, Douyin, and Kuaishou because these platforms are widely used for youth-oriented cultural content dissemination in China.

Figure 1 summarizes the two analytically separate stages of the study. The left part of the framework shows the acquisition of video, interaction, questionnaire, and interview data; the middle part shows multimodal feature extraction, leakage-controlled prediction, and SHAP interpretation; the right part shows the PLS-SEM theory-specified associative model linking digital exposure with emotional resonance, cultural identity, learning participation, and transmission intention. The video-level prediction analysis and respondent-level psychological analysis therefore remained statistically distinct.

**Figure 1:**
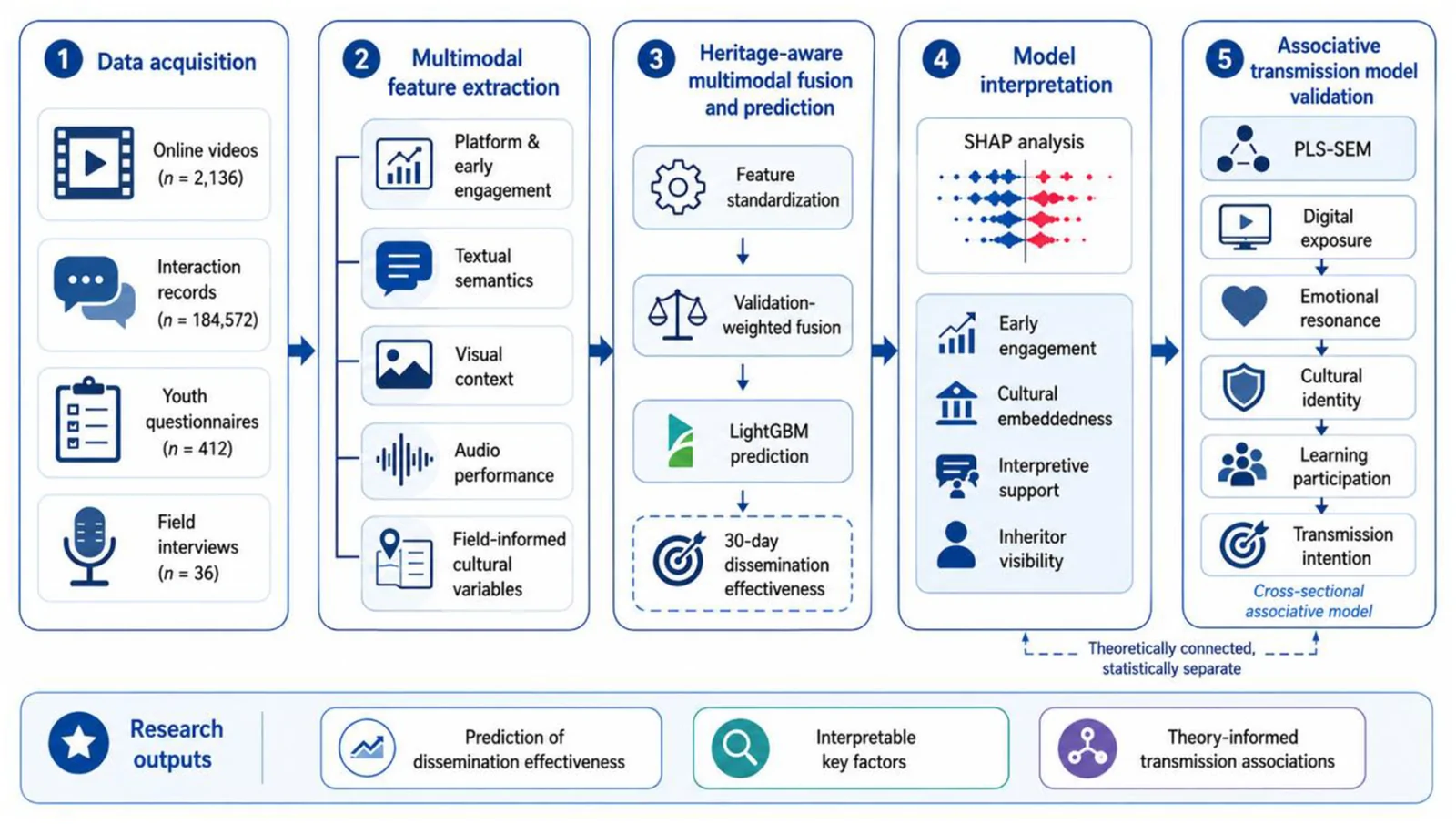
Overall research framework.

### 3.2 Online platform data collection and compliance

Online data were collected using keyword combinations related to blind ballad artists, Jindongnan narrative singing, Taihang Mountain ballad performance, local folk art, regional musical ICH, narrative singing, folk storytelling, and local quyi-related performance. The collection period covered January 2021 to December 2025. The initial retrieval returned 12,846 records. After deduplication, keyword relevance filtering, video availability checking, and manual screening, 2,136 valid videos were retained. A total of 184,572 textual interaction records were collected, including comments, bullet comments where available, hashtags, video descriptions, and early engagement records.

Only publicly accessible platform data were collected. Usernames, profile images, profile links, and other identifiable user information were removed before analysis. The study did not infer private attributes of platform users. Platform screenshots used in figures were anonymized by removing account names, profile images, user IDs, comment identities, and location indicators.

For longitudinal tracking, only videos newly uploaded during the observation period were included in the prediction dataset. The upload timestamp was recorded when the video first appeared in the platform search results or creator feed. Early engagement metrics were collected within a 24 ± 2 h window after publication, and final engagement metrics were collected within a 30 ± 2 day window. Videos for which either observation point was missed were excluded from the prediction experiment. To examine whether this exclusion introduced sample bias, the distributions of platform type, creator follower count, video length, and cultural embeddedness were compared between retained and excluded videos. No substantial imbalance was observed, as reported in Supplementary Table S1.

To avoid target leakage, the prediction model did not use final 30-day engagement variables as input. Metadata and content features were recorded at publication, early engagement indicators were recorded approximately 24 h after publication, and final engagement indicators were recorded 30 days after publication. The target variable was the normalized 30-day dissemination effectiveness score.

Figure 2 reports the screening process from the initial 12,846 platform records to the final 2,136-video analytical sample, including duplicate removal, availability checks, relevance filtering, and manual content review.

**Figure 2:**
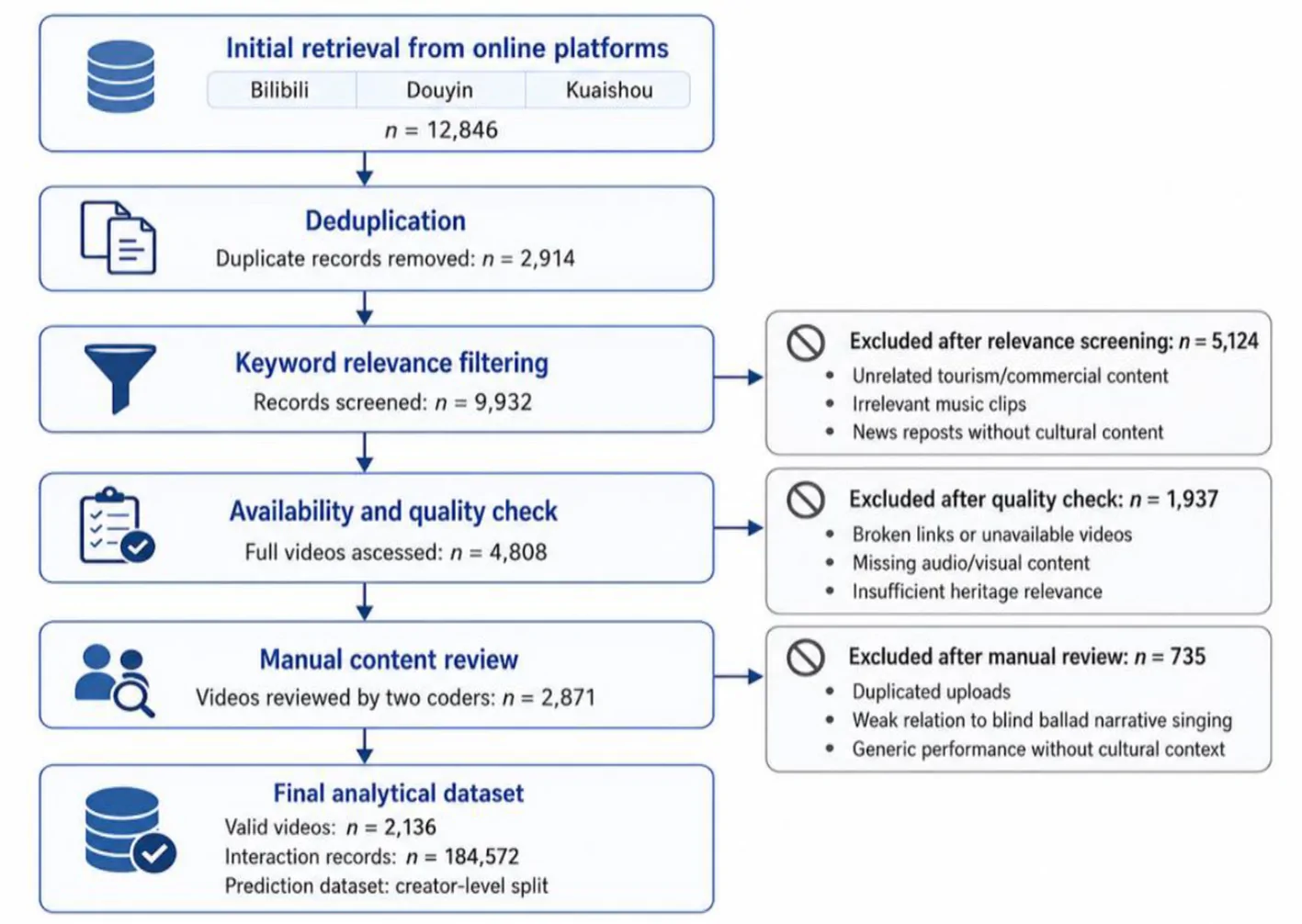
Data collection and screening flowchart.

Table 1 specifies the platforms, observation period, search terms, inclusion and exclusion rules, coder review procedure, and final online sample.

**Table 1:** *Online* data collection criteria.

Category	Criterion
Platforms	Bilibili, Douyin, Kuaishou
Time span	January 2021–December 2025
Search terms	Blind ballad artists, Jindongnan folk singing, Taihang Mountain narrative singing, local ballad performance, regional musical ICH, folk storytelling, local quyi-related narrative singing
Inclusion criteria	Videos directly related to blind ballad artists, local narrative singing, regional musical ICH, village performance, inheritance activities, or cultural explanation
Exclusion criteria	Pure tourism videos, unrelated commercial advertisements, irrelevant music clips, news reposts without cultural content, duplicated uploads, videos with inaccessible audio or visual content
Manual screening	Two coders independently reviewed relevance; disagreements were resolved by a third coder
Final sample	2,136 valid videos and 184,572 interaction records

### 3.3 Field survey and interview procedure

The field component included 36 semi-structured interviews and 412 valid youth questionnaires. Interviewees included blind ballad artists, family members, village elders, young audience members, local cultural workers, platform content producers, and community organizers. Interviews focused on transmission history, performance context, digital media use, youth participation, challenges in learning, and perceptions of online dissemination.

The questionnaire used purposive, exposure-conditioned sampling and targeted respondents aged 16–35 who had encountered related digital content or local musical heritage activities. Recruitment was conducted through the eight field sites and cultural centers, youth cultural-activity networks, and university or vocational-college youth networks associated with the study region. Because prior exposure was an eligibility condition, the questionnaire sample represents heritage-exposed youth rather than a probability sample of the general population aged 16–35.

A total of 563 potentially eligible individuals were approached or invited, 487 returned questionnaires (86.5%), and 75 responses were excluded: 26 were incomplete, 21 had abnormal completion times, and 28 showed clear straight-line response patterns. The final analytical sample therefore contained 412 valid questionnaires, corresponding to 84.6% of returned questionnaires.

The questionnaire measured digital exposure, emotional resonance, cultural identity, learning participation, and transmission intention using five-point Likert-scale items.

Three trained coders participated in the annotation. The coders had backgrounds in digital media studies, musicology, and cultural heritage research. Before formal annotation, they completed a pilot annotation session using 80 videos. During formal annotation, 300 randomly selected samples were independently checked for cultural semantic labels. The average Cohen's kappa was 0.814, indicating acceptable intercoder reliability.

The interview materials were analyzed using a codebook-based thematic procedure informed by thematic analysis (Braun and Clarke, 2006). Interviews were transcribed, anonymized, and subjected to an initial combination of deductive coding based on the research questions and inductive coding for locally specific meanings not represented in the initial categories. The first-cycle codes were consolidated into higher-order categories through coder comparison and discussion. Two coders reviewed the developing codebook, and unresolved disagreements were discussed with the third coder. The final six interviews did not generate additional higher-order categories, which was treated as an information-sufficiency criterion rather than as proof of universal thematic saturation.

The qualitative analysis ultimately organized recurring material around four higher-order themes: cultural embeddedness, accessibility, emotional resonance, and participation. Interview data were used to inform the heritage-oriented codebook and to interpret quantitative patterns; interview transcripts themselves were not entered directly as numeric predictors of video dissemination.

Table 2 summarizes the questionnaire and interview samples used in the respondent-level and qualitative components.

**Table 2:** Field survey and interview sample composition.

Sample type	Category	Number
Questionnaire respondents	Total valid responses	412
Age group	16–20	108
	21–25	147
	26–30	93
	31–35	64
Residence background	Village-origin youth	236
	Urban-only youth	176
Prior exposure	Watched related videos	331
	Participated in offline activity	118
Interviews	Blind artists	8
	Family members and village elders	9
	Youth participants	10
	Local cultural workers and creators	9

### 3.4 Variable construction

The dependent variable for the prediction task was 30-day dissemination effectiveness. Raw view counts are strongly influenced by platform recommendation mechanisms, account exposure, and upload timing. Therefore, this study used view-normalized engagement indicators to reduce the dominance of platform traffic allocation. Dissemination breadth and dissemination depth were treated as two conceptually distinct dimensions. Shares and reposts represent outward diffusion because they transfer content beyond the immediate viewing event, whereas likes, favorites, comments, and bullet comments represent progressively richer forms of audience engagement with the viewed content. These operational labels describe platform interaction patterns and should not be interpreted as direct measurements of heritage learning.

Let *V*_*i*_, *L*_*i*_, *F*_*i*_, *C*_*i*_, *S*_*i*_, *U*_*i*_, and *R*_*i*_ denote the 30-day views, likes, favorites, comments, shares, bullet comments, and reposts of video *i*, respectively. Dissemination breadth was defined as shown in Equation 1:

$$\begin{array}{l} B_{i}\;=\;log\left(1\;+\;\frac{S_{i}\;+\;R_{i}}{V_{i}\;+\;\varepsilon }\right) \end{array}$$ (1)

where *B*_*i*_ denotes the breadth of dissemination and ε is a smoothing constant. Dissemination depth was defined as shown in Equation 2:

$$\begin{array}{l} D_{i}\;=\;log\left(1\;+\;\frac{L_{i}\;+\;F_{i}\;+\;C_{i}\;+\;U_{i}}{V_{i}\;+\;\varepsilon }\right) \end{array}$$ (2)

where *D*_*i*_ denotes the depth of dissemination. The overall dissemination effectiveness score was defined as shown in Equation 3:

$$\begin{array}{l} Y_{i}\;=\;\lambda B_{i}\;+\;\left(1\;-\;\lambda \right)D_{i} \end{array}$$ (3)

where *Y*_*i*_ denotes the 30-day dissemination effectiveness score and λ controls the relative weight of dissemination breadth. The main analysis used λ = 0.35, giving slightly greater emphasis to engagement depth because regional musical ICH transmission depends not only on exposure but also on audience interpretation and participation.

The value λ = 0.35 was specified as a substantive weighting choice rather than selected to maximize predictive performance. Because the study focuses on transmission-oriented engagement rather than traffic volume alone, depth received a weight of 0.65. Sensitivity analyses were conducted at λ = 0.00, 0.20, 0.35, 0.50, and 0.65; the main predictor rankings and overall conclusions remained stable across these definitions.

To prevent target leakage, final 30-day engagement indicators were not used as model inputs. The input variables included three groups: content-intrinsic features, creator metadata, and 24-h early engagement indicators. The general input vector was defined as shown in Equation 4:

$$\begin{array}{l} x_{i}\;=\;c_{i}\;⊕\;m_{i}\;⊕\;e_{i}^{24h} \end{array}$$ (4)

where *c*_*i*_ denotes content features, *m*_*i*_ denotes creator and upload metadata, $e_{i}^{24h}$ denotes 24-hour early-stage engagement indicators, and ⊕ denotes vector concatenation.

### 3.5 Heritage-oriented multimodal feature representation

The feature system included five groups: platform metadata and early engagement features, textual semantic features, visual contextual features, audio performance features, and field-informed cultural features. Table 3 summarizes the operational variables and extraction procedures for each modality.

**Table 3:** Feature groups and operational definitions.

Feature group	Symbol	Representative variables	Extraction method
Metadata and early engagement	*h* ^*P*^	Creator followers, upload time, video length, platform type, 24-h engagement ratios	Platform metadata and early observation window
Textual semantics	*h* ^*T*^	Cultural keywords, narrative clarity, youth-oriented interpretive support, sentiment, topic distribution, dialect markers	Transformer embeddings, topic modeling, dictionary matching, manual verification
Visual context	*h* ^*V*^	Inheritor visibility, village scene salience, instrument visibility, subtitle visibility, audience presence	Key-frame extraction and CLIP-based prompt matching
Audio performance	*h* ^*A*^	Singing–speech ratio, pitch variation, rhythm density, timbre embedding, accompaniment strength	HuBERT embeddings and acoustic descriptors
Field-informed cultural variables	*h* ^*F*^	Cultural embeddedness, community context, transmission relevance, interpretive accessibility	Predefined codebook informed by field interviews and expert coding

The principal heritage-specific construct was termed cultural embeddedness throughout the analysis. It refers to the extent to which observable video content locates the performance within recognizable performer, linguistic, repertoire, instrumental, spatial, explanatory, and transmission contexts. It does not constitute an essentialist judgment of whether a performance is “authentic” as shown in Equation 5.

$$\begin{array}{l} CE_{i}\;=\;\overset{7}{\underset{k\;=\;1}{\sum }}w_{k}\;z_{ik},\;\;\;\;\overset{7}{\underset{k\;=\;1}{\sum }}w_{k}\;=\;1 \end{array}$$ (5)

where *CE*_*i*_ denotes the cultural embeddedness score of video *i*, *z*_*ik*_ denotes the binary or standardized score of indicator *k*, and the main analysis used equal weights *w*_*k*_ = 1/7. As a robustness check, an expert-weighted score was also calculated, and the ranking of key predictors remained stable.

Table 4 defines each cultural-embeddedness indicator from observable video evidence.

**Table 4:** Coding rubric for cultural embeddedness.

Indicator	Score = 1	Score = 0
Inheritor visibility	A clearly identifiable performer or inheritor appears in the video	No performer or inheritor is visible
Village or folk scene	Village courtyard, local ritual, rural gathering, or community setting is visible	Generic stage, studio, or unrelated background
Dialect or local accent	Dialectal singing or narration is audible or explicitly subtitled	Mandarin-only content or no identifiable local speech
Traditional repertoire or tune	A local tune, repertoire, or narrative form is named or recognizable	No repertoire or traditional form is identifiable
Traditional instrument	Local accompaniment or traditional instrument is visible or audible	No instrument or only generic background music
Cultural explanation	The video explains history, lyrics, repertoire, artist, or custom	No cultural explanation
Transmission scene	Family, apprenticeship, village teaching, or community participation appears	No transmission-related scene

The field interviews informed the observable categories in this rubric but were not directly transformed into dissemination outcomes. The codebook was finalized before the main predictive interpretation. During video-level cultural coding, the coders did not have access to the 30-day dissemination score, model output, or SHAP ranking. This separation reduced circularity between cultural-variable construction and interpretation of predictive importance.

Youth-oriented interpretive support was measured as shown in Equation 6:

$$\begin{array}{l} YIS_{i}\;=\;\overset{5}{\underset{k\;=\;1}{\sum }}q_{k}\;u_{ik},\;\;\;\;\overset{5}{\underset{k\;=\;1}{\sum }}q_{k}\;=\;1 \end{array}$$ (6)

where *YIS*_*i*_ denotes youth-oriented interpretive support, *u*_*ik*_ denotes the standardized score of subtitles, simplified cultural explanation, story-based introduction, interactive questioning, or modern contextualization, and the main analysis used equal weights *q*_*k*_ = 1/5.

Textual content was encoded using a Chinese transformer encoder. For video *i*, the title, description, subtitles, hashtags, and early comments were combined into a document *d*_*i*_. The textual representation was obtained as shown in Equation 7.

$$\begin{array}{l} h_{i}^{T}\;=\;f_{T}\left(d_{i};\;\theta_{T}\right) \end{array}$$ (7)

where *f*_*T*_ denotes the text encoder and θ_*T*_ denotes its parameters. Topic features were additionally derived using unsupervised topic modeling and manual topic merging, following recent discussions on topic modeling for online community and qualitative data analysis (Kaur and Wallace, 2024).

The text encoder checkpoint was hfl/chinese-roberta-wwm-ext. Only comments observed within the 24-h early window were eligible for prediction features. URLs, account identifiers, and user mentions were removed; emojis were normalized, stop words were filtered, and dialect terms were retained. Up to 200 early comments per video were used. When more than 200 comments were available, comments were sampled across the 24-h window to avoid concentrating the representation in a single short engagement burst.

Visual features were extracted from 12 uniformly sampled key frames per video. A CLIP-based image-text matching strategy was used to measure similarity between video frames and heritage-related visual prompts, such as “blind folk artist performing,” “traditional instrument,” “village courtyard performance,” “local ritual scene,” and “young audience watching” (Radford et al., 2021). The visual representation was calculated as shown in Equation 8.

$$\begin{array}{l} h_{i}^{V}\;=\;\frac{1}{12}\;\overset{12}{\underset{j\;=\;1}{\sum }}f_{V}\left(I_{ij};\;\theta_{V}\right) \end{array}$$ (8)

The visual checkpoint was openai/clip-vit-base-patch32. The predefined prompt set was: “blind folk artist performing,” “traditional instrument,” “village courtyard performance,” “local ritual scene,” “young audience watching,” “family teaching traditional singing,” “subtitled folk performance,” and “formal stage performance.” Prompt definitions were fixed before model evaluation.

where *I*_*ij*_ denotes key frame *j*, and *f*_*V*_ denotes the visual encoder.

Audio tracks were resampled to 16 kHz and processed using HuBERT embeddings and acoustic descriptors (Hsu et al., 2021). The audio representation was defined as shown in Equation 9.

$$\begin{array}{l} h_{i}^{A}\;=\;f_{H}\left(a_{i};\;\theta_{H}\right)\;⊕\;g\left(a_{i}\right) \end{array}$$ (9)

where *a*_*i*_ denotes the audio track, *f*_*H*_ denotes the HuBERT encoder, and *g*(*a*_*i*_) denotes acoustic features including pitch variance, rhythm density, energy variance, and singing–speech ratio. For videos with continuous performance segments longer than 20 s, VideoMAE features were additionally used to capture temporal performance patterns (Tong et al., 2022). The questionnaire constructs and representative items are provided in Supplementary Table S6.

The HuBERT checkpoint was facebook/hubert-base-ls960. Audio preprocessing was deliberately conservative: each track was converted to mono, resampled to 16 kHz, corrected for DC offset, and peak-normalized before windowing. No neural source separation or aggressive spectral denoising was applied because these procedures could alter dialectal speech cues, vocal timbre, instrumental harmonics, and singing–speech structure that were themselves heritage-relevant features. For videos with continuous performance segments longer than 20 s, VideoMAE features were extracted using the MCG-NJU/videomae-base checkpoint.

### 3.6 Heritage-aware explainable dissemination framework

The proposed Heritage-aware Explainable Dissemination Framework integrates multimodal feature extraction, validation-weighted feature fusion, LightGBM prediction, and SHAP-based interpretation. It is not intended as a new end-to-end neural architecture. Its methodological contribution lies in adapting multimodal AI representations to the cultural characteristics of regional musical ICH, controlling target leakage in dissemination prediction, and producing interpretable results that can be linked to field-based transmission evidence.

Considering the moderate size of the dataset and the heterogeneous nature of multimodal features, LightGBM was adopted as the final prediction learner rather than an end-to-end deep neural architecture (Ke et al., 2017). This choice reduced the risk of overfitting and allowed robust integration of structured platform variables, semantic features, audio descriptors, visual indicators, and field-informed cultural variables. XGBoost (Chen and Guestrin, 2016) and TabNet (Arik and Pfister, 2021) were used as comparative models.

Each modality-specific feature vector was standardized and projected into a comparable feature space through normalization and dimensionality reduction. High-dimensional text, visual, audio, and temporal embeddings were reduced using principal component analysis fitted exclusively on the training fold. Components were retained until 95% of training-set variance was explained, with a maximum of 128 components for any modality. The fitted transformation was then applied unchanged to validation and test data. The fused representation of video was defined as Equation 10:

$$\begin{array}{l} z_{i}\;=\;\alpha_{P}\;h_{i}^{P}\;⊕\;\alpha_{T}\;h_{i}^{T}\;⊕\;\alpha_{V}\;h_{i}^{V}\;⊕\;\alpha_{A}\;h_{i}^{A}\;⊕\;\alpha_{F}\;h_{i}^{F} \end{array}$$ (10)

where $h_{i}^{P}$, $h_{i}^{T}$, $h_{i}^{V}$, $h_{i}^{A}$, and $h_{i}^{F}$ denote platform-side, textual, visual, audio, and field-informed feature vectors, respectively. The modality weights satisfy (Equation 11):

$$\begin{array}{l} \alpha_{P}\;+\;\alpha_{T}\;+\;\alpha_{V}\;+\;\alpha_{A}\;+\;\alpha_{F}\;=\;1,\;\;\;\alpha_{m}\;\geq \;0 \end{array}$$ (11)

The modality weights were selected on the validation set through grid search. Weights were searched in increments of 0.05 under the sum-to-one constraint. The selected setting was α_*P*_ = 0.30, α_*T*_ = 0.25, α_*V*_ = 0.20, α_*A*_ = 0.10, and α_*F*_ = 0.15. No test-set information was used in weight selection. This design avoids unstable end-to-end training on a moderate-sized dataset and allows the fused representation to be directly used by LightGBM.

The dissemination score was predicted as Equation 12:

$$\begin{array}{l} Y_{i}\;=\;f_{\text{LGBM}}\left(z_{i};\;\Theta \right) \end{array}$$ (12)

where *f*_LGBM_ denotes the LightGBM prediction function and Θ denotes model parameters. The training objective was Equation 13:

$$\begin{array}{l} L\left(\Theta \right)\;=\;\frac{1}{N}\;\overset{N}{\underset{i\;=\;1}{\sum }}{\left(Y_{i}\;-\;Y_{i}\right)}^{2}\;+\;\eta \Omega \left(\Theta \right) \end{array}$$ (13)

where *N* is the number of training samples and η is the regularization coefficient.

Model interpretation was performed using SHAP (SHapley Additive exPlanations) (Lundberg and Lee, 2017). For each prediction, the model output was decomposed as Equation 14:

$$\begin{array}{l} Y_{i}\;=\;\varphi_{0}\;+\;\overset{M}{\underset{j\;=\;1}{\sum }}\varphi_{ij} \end{array}$$ (14)

where φ_0_ is the expected model output, *M* is the number of features, and φ_*ij*_ is the contribution of feature *j* to sample *i*.

SHAP values were used strictly as explanations of predictive model output. They were not interpreted as causal or intervention effects.

Figure 3 summarizes modality-specific extraction, training-fold dimensionality reduction, validation-weighted fusion, LightGBM prediction, and *post hoc* SHAP interpretation.

**Figure 3:**
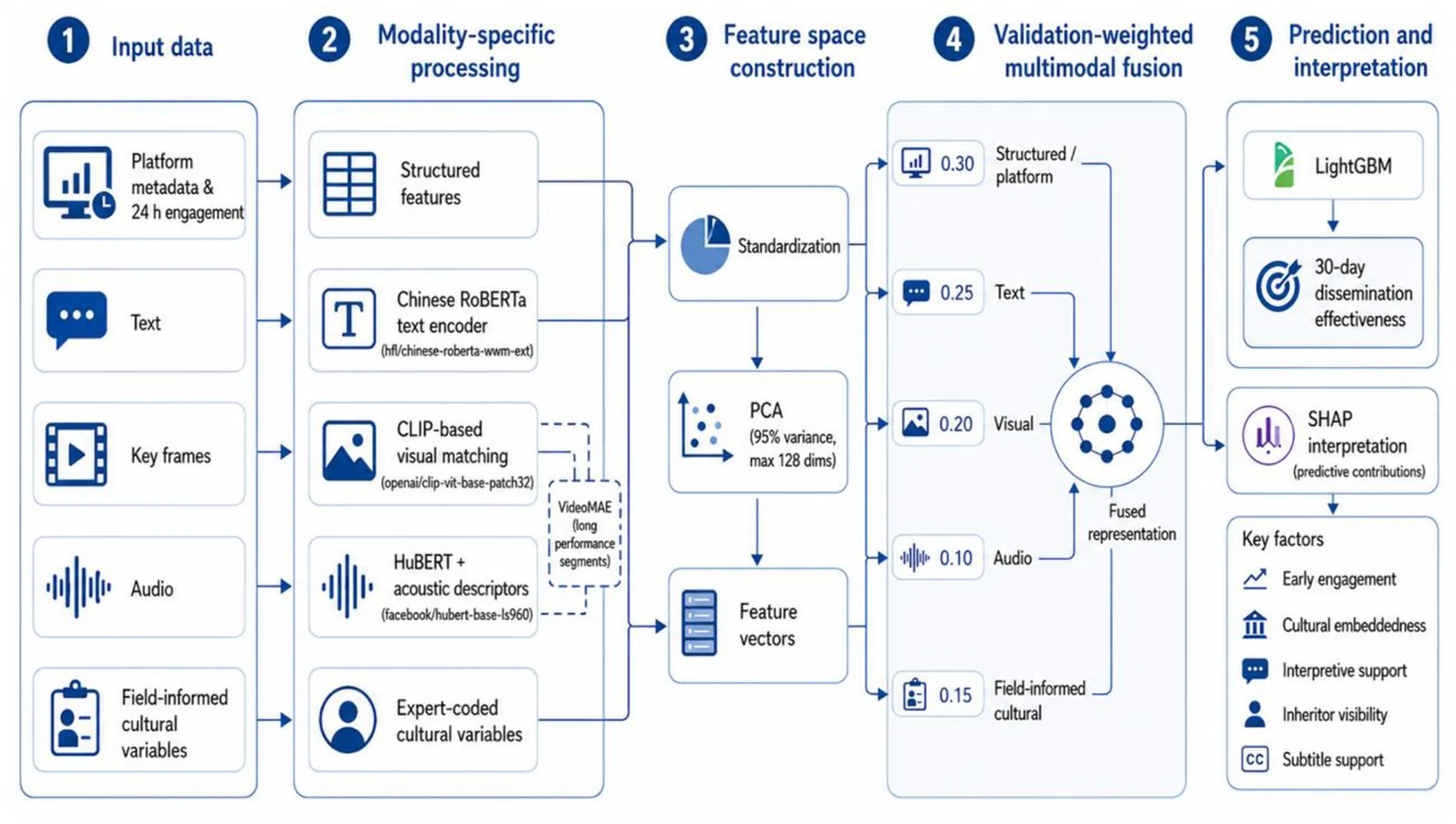
Architecture of the Heritage-aware Explainable Dissemination Framework.

### 3.7 Structural equation model for transmission mechanism

To examine the theory-specified statistical relationships from digital exposure to intergenerational transmission intention, partial least squares structural equation modeling (PLS-SEM) was used (Hair et al., 2022). The latent variables were digital exposure (DE), emotional resonance (ER), cultural identity (CI), learning participation (LP), and transmission intention (TI). Bootstrapping with 5,000 resamples was used to estimate path significance and confidence intervals.

PLS-SEM was selected because the respondent-level analysis was prediction- and explanation-oriented and focused on estimating a set of latent-variable associations and indirect paths alongside the computational prediction component. The choice was not based on an assumption that covariance-based SEM would be impossible with the available sample. Rather, PLS-SEM provided latent scores, was compatible with the study's prediction-oriented analytical emphasis, and permitted nonparametric bootstrap inference without requiring multivariate normality.

The structural model was specified as shown in Equation 15:

$$\begin{array}{l} \eta =B\eta +\Gamma \xi +\zeta \end{array}$$ (15)

where **η** denotes endogenous latent variables, **ξ** denotes exogenous latent variables, **B** denotes relationships among endogenous latent variables, **Γ** denotes associations from exogenous to endogenous variables, and **ζ** denotes residual terms.

The measurement model was specified by Equations 16 and 17:

$$\begin{array}{l} x=\Lambda_{x}\xi +\delta \end{array}$$ (16)

$$\begin{array}{l} y=\Lambda_{y}\eta +\varepsilon \end{array}$$ (17)

where **x** and **y** denote observed indicators, **Λ**_*x*_ and **Λ**_*y*_ denote factor loading matrices, and **δ** and **ε** denote measurement errors. The main hypothesized pathway was:

$$\begin{array}{l} DE\to ER\to CI\to LP\to TI \end{array}$$ (18)

Equation 18 represents a directional statistical specification derived from the hypotheses; it does not by itself establish temporal ordering. Reciprocal relationships, reverse associations, or omitted common antecedents cannot be excluded by the cross-sectional design.

Sample-size adequacy was assessed using the inverse square-root approach for PLS-SEM (Kock and Hadaya, 2018). At a 5% significance level and 80% power, the minimum sample size was evaluated using the following expression: *N*_min_ > (2.486/|β_min_|) . For a prespecified minimum substantive path magnitude of |β| = 0.15, the resulting minimum sample size is approximately 275. The analytical sample of 412 therefore exceeded this threshold for the core structural relationships. Very small direct associations, including the observed DE → TI coefficient, are nevertheless interpreted cautiously rather than treated as evidence that the sample guarantees high power for every possible weak association.

Common method bias was examined using Harman's single-factor test and full collinearity variance inflation factor (VIF). The first unrotated factor explained 31.8% of the total variance, below the 50% threshold. Full collinearity VIF values ranged from 1.214 to 2.306, below the conservative threshold of 3.3, indicating that common method bias was unlikely to dominate the results. Measurement quality was additionally evaluated using individual outer loadings, Cronbach's α, composite reliability, average variance extracted, Fornell–Larcker criteria, HTMT ratios, and SRMR. These statistics are reported in the main Results section rather than being available only in supplementary material.

### 3.8 Implementation details

All data processing and model training were implemented in Python 3.10. The software environment consisted of PyTorch 2.2.2, Transformers 4.41.2, scikit-learn 1.4.2, LightGBM 4.3.0, XGBoost 2.0.3, SHAP 0.45.1, and librosa 0.10.2.post1. Text cleaning included duplicate removal, emoji normalization, stop-word filtering, dialect term preservation, and domain dictionary matching. The text encoder used a maximum sequence length of 256 tokens. For long comment sequences, sentence-level embeddings were averaged.

Visual features were extracted from 12 key frames per video. Frames were resized to 224 × 224 pixels. Audio was resampled to 16 kHz. HuBERT embeddings were extracted from 20-s audio windows and averaged for long videos. Acoustic descriptors included 13 Mel-frequency cepstral coefficients, pitch mean, pitch standard deviation, energy variance, zero-crossing rate, and rhythm density. Before HuBERT processing, audio was converted to mono, DC offset was removed, and peak amplitude was normalized. No aggressive denoising or learned source-separation model was used.

The dataset was split into training, validation, and test sets at a ratio of 70:15:15. To reduce leakage from creator-level similarity, all splits were performed at the creator level, ensuring that videos from the same creator did not appear in both training and test sets. Five repeated creator-level splits were conducted using the fixed random seeds 17, 29, 43, 71, and 101. All preprocessing transformations requiring fitted parameters, including standardization and PCA, were estimated within the corresponding training data and subsequently applied to validation and test data.

LightGBM used 600 estimators, learning rate 0.035, maximum depth 7, 48 leaves, feature fraction 0.85, bagging fraction 0.80, and early stopping with 60 validation rounds. XGBoost used 500 estimators, maximum depth 6, learning rate 0.04, subsample 0.85, and column sample by tree 0.80. TabNet used decision dimension 32, attention dimension 32, five decision steps, sparsity regularization 1 × 104, batch size 128, and Adam optimizer with learning rate 2 × 10.

## 4 Experiments

### 4.1 Experimental objectives

The experiments were designed to answer four research questions: **RQ1:** Can multimodal AI features predict the 30-day dissemination effectiveness of regional musical ICH videos without relying on final engagement indicators? **RQ2:** Which feature groups contribute most to dissemination prediction? **RQ3:** Which cultural and digital features are most influential according to model interpretation? **RQ4:** Is digital exposure associated with intergenerational transmission intention through emotional resonance, cultural identity, and learning participation?

### 4.2 Experimental design

Table 5 summarizes the empirical validation logic and corresponding result sections.

**Table 5:** Experimental design and validation logic.

Experiment	Validation purpose	Corresponding result
Model comparison	Comparative predictive performance	Table 6
Feature-group ablation	Incremental contribution of multimodal features	Table 7
Robustness checks	Stability across outcome definitions and settings	Table 9, Supplementary Table S3
Early-engagement removal	Dependence on early popularity signals	Table 8
SHAP explanation	Predictive contribution and direction of major features	Table 10, Figure 5
PLS-SEM analysis	Measurement quality and respondent-level associative pathways	Tables 11–14, Figure 6

### 4.3 Baselines

The proposed framework was compared with seven baselines:

1. Ridge regression: a linear statistical baseline using metadata and early engagement features.
2. Random forest: a nonlinear ensemble baseline.
3. XGBoost: a scalable tree boosting baseline (Chen and Guestrin, 2016).
4. LightGBM: a gradient boosting decision tree baseline (Ke et al., 2017).
5. TabNet: an attentive interpretable tabular learning model (Arik and Pfister, 2021).
6. Text-only transformer: a text-only model using title, description, subtitles, and early comments.
7. Concatenation-MLP: a multimodal neural baseline that directly concatenates all modality embeddings and feeds them into a multilayer perceptron.

The proposed framework differs from the baselines by using heritage-oriented multimodal feature construction, validation-weighted multimodal fusion, leakage-controlled input design, creator-level splitting, and SHAP-based interpretation.

### 4.4 Evaluation metrics

For regression prediction, mean absolute error (MAE), root mean square error (RMSE), coefficient of determination (R2), and Spearman correlation coefficient were calculated using Equations 19–22:

$$\begin{array}{l} \text{MAE}=\frac{1}{N}\overset{N}{\underset{i=1}{\sum }}|Y_{i}-{Ŷ}_{i}| \end{array}$$ (19)

$$\begin{array}{l} \text{RMSE}=\sqrt{\frac{1}{N}\overset{N}{\underset{i=1}{\sum }}{\left(Y_{i}-{Ŷ}_{i}\right)}^{2}} \end{array}$$ (20)

$$\begin{array}{l} R^{2}=1-\frac{\sum_{i=1}^{N}{\left(Y_{i}-{Ŷ}_{i}\right)}^{2}}{\sum_{i=1}^{N}{\left(Y_{i}-Ȳ\right)}^{2}} \end{array}$$ (21)

$$\begin{array}{l} \rho =1-\frac{6\sum_{i=1}^{N}d_{i}^{2}}{N\left(N^{2}-1\right)} \end{array}$$ (22)

where *Y*_*i*_ is the observed dissemination score, Ŷ_*i*_ is the predicted dissemination score, Ȳ is the mean observed score, and *d*_*i*_ is the rank difference between observed and predicted scores.

For the high-dissemination classification analysis, videos in the top 30% of *Y*_*i*_ were labeled as high-dissemination videos. Precision, recall, and F1-score were calculated using Equations 23–25:

$$\text{Precision}=\frac{\text{TP}}{\text{TP}+\text{FP}}$$ (23)

$$\text{Recall}=\frac{\text{TP}}{\text{TP}+\text{FN}}$$ (24)

$$\text{F}1=\frac{2\times \text{Precision}\times \text{Recall}}{\text{Precision}+\text{Recall}}$$ (25)

where *TP*, *FP*, and *FN* denote true positives, false positives, and false negatives, respectively.

### 4.5 Ablation, robustness, and statistical comparison

Ablation experiments were conducted by gradually adding feature groups: metadata and early engagement only, text only, visual only, audio only, metadata plus text, metadata plus text and visual features, metadata plus text, visual, and audio features, and the full feature set. Additional core-feature ablation experiments removed early engagement, cultural embeddedness, youth-oriented interpretive support, visual context, and audio features. Robustness checks included five repeated creator-level splits, random split comparison, platform-specific experiments, sensitivity analysis of the dissemination weight, and expert-weighted cultural embeddedness score comparison. All model comparisons were conducted under creator-level splits. This setting is stricter than random splitting because it prevents videos from the same creator from appearing in both training and test sets. Because results from repeated train/test partitions are not statistically independent, conventional paired *t*-tests across the five splits were not used for the principal inferential comparison. Instead, the corrected resampled t procedure was applied to paired split-level differences following the variance correction for overlapping training and test samples described by Nadeau and Bengio (Nadeau and Bengio, 2003). For a 70% training and 15% test fraction, the correction factor was:

$$\begin{array}{l} 1/5\;+\;0.15/0.70\;=\;0.4143 \end{array}$$

Corrected 95% confidence intervals and Hedges *g*_*av*_ were reported together with *p* values. Given the small number of repeated splits, these tests were interpreted as supporting evidence rather than as the sole basis for comparative model performance.

## 5 Results

### 5.1 Descriptive statistics

The final dataset contained 2,136 valid videos. Among them, 38.2% were collected from Bilibili, 34.7% from Douyin, and 27.1% from Kuaishou. The median video length was 126 s, and 68.4% of videos were shorter than 5 min. Videos with subtitles accounted for 54.7%. Identifiable inheritors appeared in 41.3% of videos. Village or community scenes appeared in 36.8% of videos, while stage or formal performance scenes appeared in 29.5%. Videos containing explicit youth-oriented interpretive support accounted for 31.6%.

The 30-day dissemination effectiveness score showed a right-skewed distribution. After view-normalized calculation and logarithmic transformation, the distribution became more stable, although a small right tail remained. This pattern is consistent with platform-based cultural content dissemination, where a limited number of videos receive disproportionately strong engagement.

Figure 4 shows the right-skewed distribution of 30-day dissemination effectiveness after view normalization and logarithmic transformation. Most observations were concentrated in the middle range, with a limited residual right tail. Detailed descriptive statistics are reported in Supplementary Table S2.

**Figure 4:**
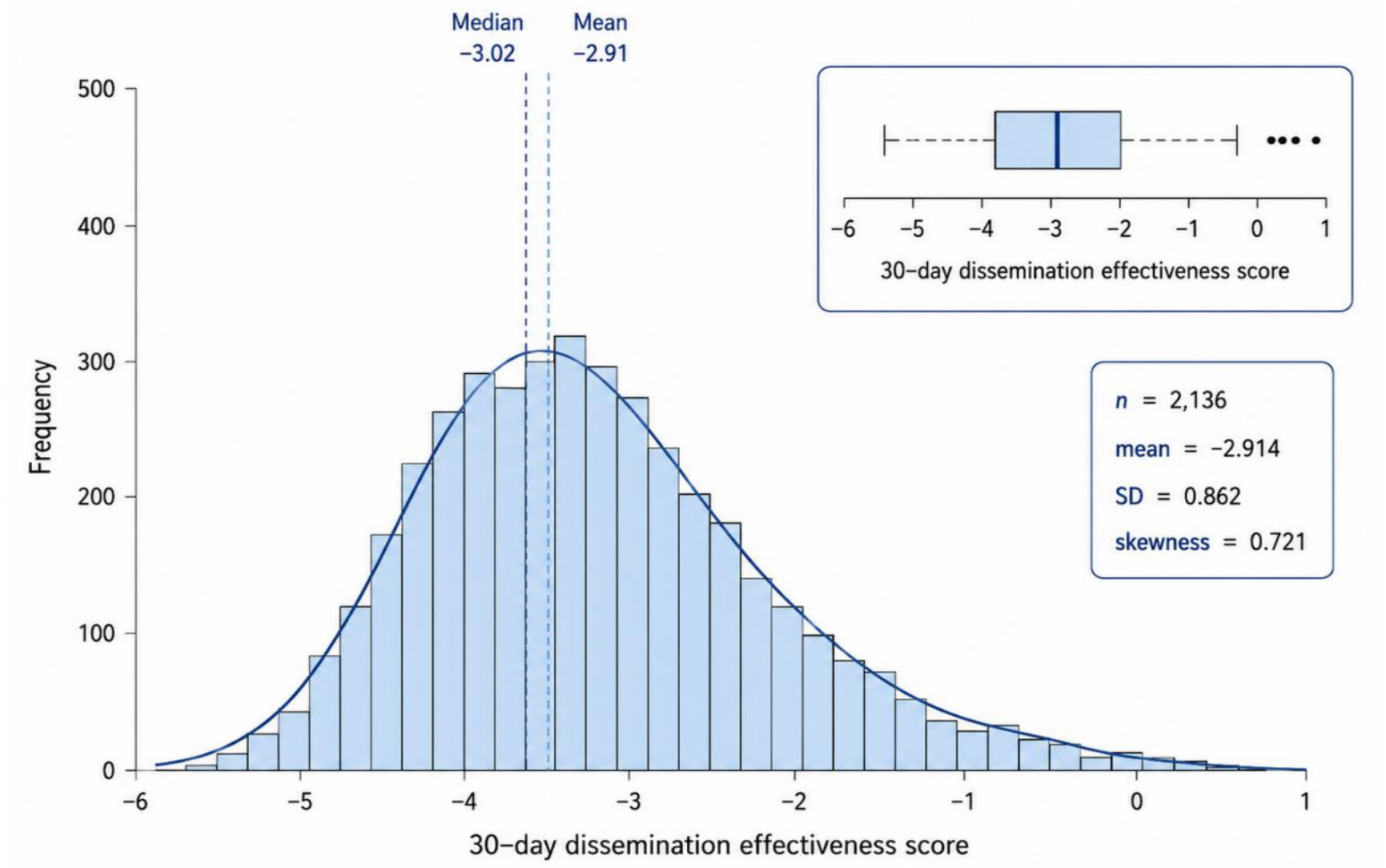
Distribution of 30-day dissemination effectiveness.

### 5.2 Prediction performance

Table 6 compares the prediction performance of all models across five creator-level splits. The proposed framework achieved the best overall performance, with MAE = 0.412 ± 0.014, RMSE = 0.552 ± 0.018, *R*^2^ = 0.706 ± 0.020, Spearman's ρ = 0.781 ± 0.019, and F1-score = 0.784 ± 0.021. Relative to the strongest non-framework baseline, Concatenation-MLP, the proposed framework reduced RMSE by 0.017. The Nadeau–Bengio corrected comparison yielded a 95% CI of [0.005, 0.029], *p* = 0.015, and Hedges *g*_*av*_ = 0.81. The corresponding *R*^2^ difference was 0.023, with a corrected 95% CI of [0.008, 0.038], *p* = 0.014, and *g*_*av*_ = 0.97. These results support a moderate and consistent advantage while acknowledging uncertainty associated with the limited number of repeated creator-level splits.

**Table 6:** Model comparison for 30-day dissemination effectiveness prediction.

Model	Input setting	MAE ↓	RMSE ↓	*R*^2^↑	Spearman's *ρ↑*	*F*1 ↑
Ridge regression	Metadata + early engagement	0.548 ± 0.020	0.718 ± 0.026	0.431 ± 0.034	0.604 ± 0.028	0.629 ± 0.030
Random forest	Structured features	0.482 ± 0.018	0.636 ± 0.023	0.593 ± 0.027	0.692 ± 0.024	0.708 ± 0.026
XGBoost	Structured + semantic features	0.456 ± 0.016	0.604 ± 0.020	0.642 ± 0.025	0.729 ± 0.022	0.742 ± 0.024
LightGBM	Structured + semantic features	0.441 ± 0.015	0.586 ± 0.019	0.665 ± 0.023	0.744 ± 0.021	0.759 ± 0.023
TabNet	Full structured features	0.433 ± 0.017	0.578 ± 0.021	0.674 ± 0.026	0.752 ± 0.023	0.762 ± 0.025
Text-only transformer	Text features only	0.469 ± 0.019	0.618 ± 0.025	0.625 ± 0.029	0.711 ± 0.025	0.727 ± 0.028
Concatenation-MLP	Direct multimodal concatenation	0.426 ± 0.016	0.569 ± 0.020	0.683 ± 0.023	0.760 ± 0.021	0.771 ± 0.022
Proposed framework	Heritage-aware multimodal fusion	0.412 ± 0.014	0.552 ± 0.018	0.706 ± 0.020	0.781 ± 0.019	0.784 ± 0.021

The results indicate that content and cultural features provide additional explanatory value beyond early engagement and creator metadata. The advantage of the proposed framework was moderate but consistent across all five creator-level splits. This is important because the creator-level split is stricter than random splitting and reduces the risk of learning creator-specific patterns rather than generalizable content features. Ridge regression performed the weakest, suggesting that dissemination effectiveness was not adequately explained by linear relationships. Tree-based models performed better, confirming the importance of nonlinear feature interactions. The text-only model achieved reasonable performance, indicating that titles, subtitles, descriptions, and early comments contained meaningful cultural and emotional signals. However, it was less effective than full multimodal models, demonstrating that regional musical ICH videos require visual and audio contextual information for more accurate prediction.

### 5.3 Feature-group ablation analysis

Table 7 reports the feature-group ablation results. Metadata and early engagement features alone achieved *R*^2^ = 0.655. Textual semantic features alone achieved *R*^2^ = 0.612, indicating that textual information provided a substantial but incomplete representation. Visual-only and audio-only models achieved lower *R*^2^ values, but both contributed positively when combined with other modalities. The full feature set achieved *R*^2^ = 0.706.

**Table 7:** Feature-group ablation analysis.

Feature combination	MAE ↓	RMSE ↓	*R*^2^↑	Spearman's *ρ↑*
Metadata + early engagement only	0.448 ± 0.016	0.594 ± 0.020	0.655 ± 0.024	0.736 ± 0.022
Text only	0.476 ± 0.019	0.629 ± 0.024	0.612 ± 0.029	0.704 ± 0.025
Visual only	0.521 ± 0.021	0.684 ± 0.027	0.541 ± 0.034	0.651 ± 0.030
Audio only	0.536 ± 0.023	0.701 ± 0.029	0.518 ± 0.036	0.628 ± 0.032
Metadata + text	0.432 ± 0.015	0.574 ± 0.019	0.678 ± 0.023	0.755 ± 0.021
Metadata + text + visual	0.421 ± 0.014	0.561 ± 0.018	0.692 ± 0.021	0.769 ± 0.020
Metadata + text + visual + audio	0.416 ± 0.014	0.557 ± 0.018	0.699 ± 0.021	0.776 ± 0.019
Full features	0.412 ± 0.014	0.552 ± 0.018	0.706 ± 0.020	0.781 ± 0.019

The largest improvement came from adding textual semantic features to metadata and early engagement. Visual features produced a moderate improvement, especially for videos containing identifiable inheritors, village scenes, instruments, and community performance environments. Audio features produced a smaller but stable gain. The relatively weak performance of audio-only features may be related to background noise, inconsistent audio quality, and music compression in short videos. Nevertheless, audio features improved model stability in videos where textual descriptions were sparse.

### 5.4 Core-feature, leakage-control, and outcome-definition robustness

To further test whether the model merely relied on early popularity signals, a core-feature ablation analysis was conducted. Table 8 reports the results. Removing 24-h early engagement reduced *R*^2^ from 0.706 to 0.648, confirming that early engagement contributed substantially to prediction. However, the model without early engagement still retained meaningful predictive capacity, indicating that content and heritage-oriented features had independent predictive value.

**Table 8:** Leakage-control and core-feature ablation.

Model setting	MAE ↓	*R*^2^ ↑	Change in *R*^2^
Full framework	0.412	0.706	–
Without 24-h early engagement	0.461	0.648	−0.058
Without cultural embeddedness score	0.434	0.682	−0.024
Without youth-oriented interpretive support	0.438	0.687	−0.019
Without visual context features	0.426	0.692	−0.014
Without audio features	0.419	0.699	−0.007
Content-only framework	0.472	0.631	−0.075

The decrease caused by removing cultural embeddedness was larger than that caused by removing visual context or audio features alone. This suggests that cultural embeddedness functions as a cross-modal construct rather than a single visual or textual signal. It integrates inheritor presence, local scene, dialectal expression, traditional repertoire, instruments, and transmission context. Removing youth-oriented interpretive support also reduced performance. The content-only model achieved *R*^2^ = 0.631, which was lower than the full model but still higher than several baselines.

Because dissemination effectiveness is a constructed outcome, sensitivity analysis was conducted across alternative breadth/depth weights. Table 9 shows that predictive performance changed only moderately across λ = 0.00 to 0.65. The main setting λ = 0.35 produced the highest *R*^2^, but the difference from equal weighting was small, and the major predictor rankings remained stable. This indicates that the substantive conclusions were not dependent on a single narrow outcome definition.

**Table 9:** Sensitivity of predictive performance to the dissemination breadth weight.

λ for breadth	Depth weight	MAE ↓	RMSE ↓	Association with model output
0.00	1.00	0.423	0.566	0.688
0.20	Cultural embeddedness score	0.416	0.558	0.699
0.35	0.65	0.412	0.552	0.706
0.50	0.50	0.415	0.556	0.701
0.65	0.35	0.421	0.563	0.691

### 5.5 Explainability analysis

SHAP analysis was conducted to interpret the model outputs. Figure 5 presents the SHAP summary plot, and Table 10 reports the top 15 features ranked by mean absolute SHAP value.

**Figure 5:**
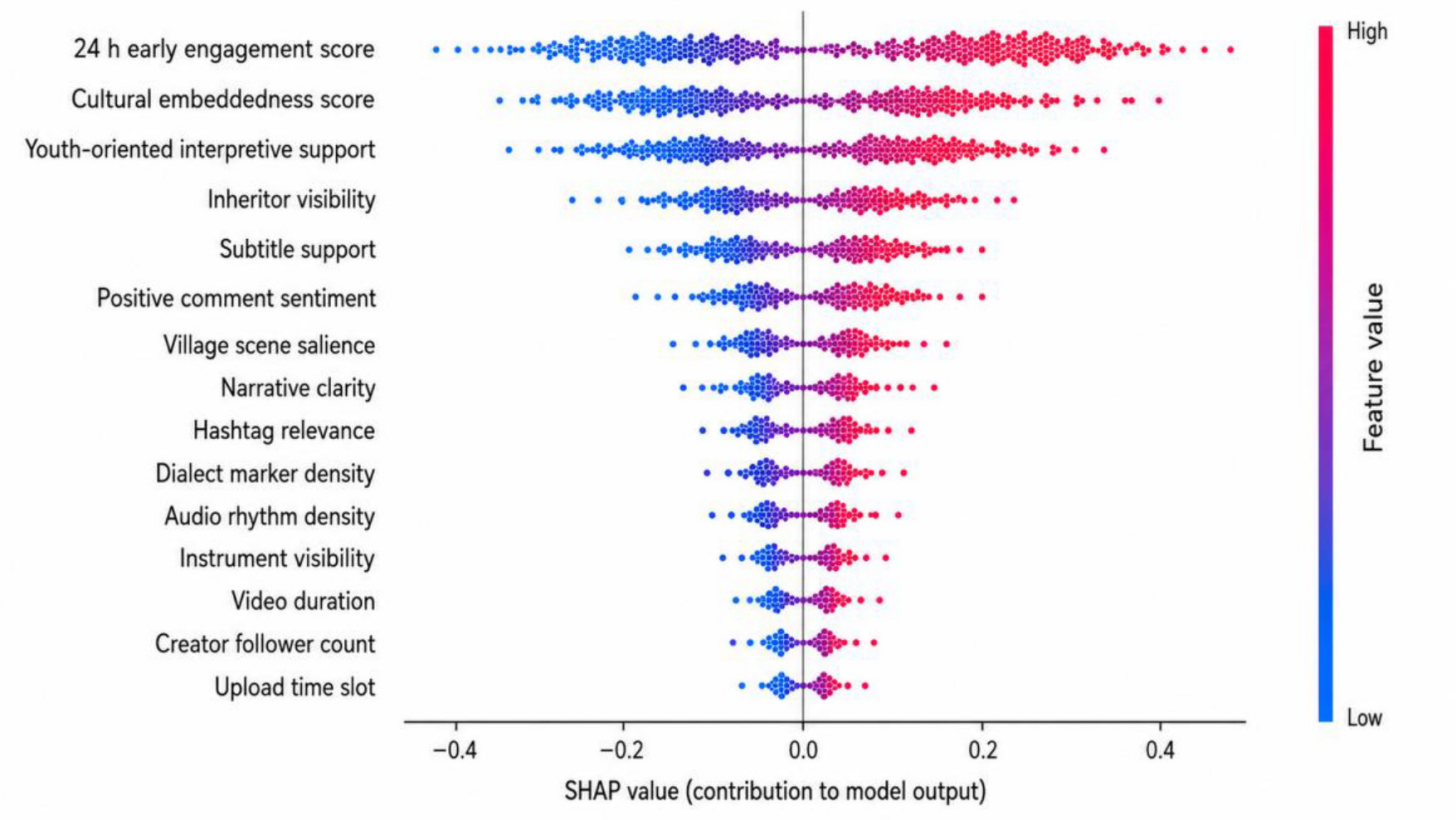
SHAP summary plot of the proposed framework.

**Table 10:** Top features influencing 30-day dissemination effectiveness prediction.

Rank	Feature	Feature group	Mean |*SHAP*|	Hypothesis/result
1	24-h early engagement score	Early engagement	0.154	H1 supported
2	Cultural authenticity/embeddedness score	Heritage semantic	0.146	H2 supported
3	Youth-oriented interpretive support	Textual semantic	0.132	H3 supported
4	Inheritor visibility	Visual/cultural	0.108	H4 supported
5	Subtitle support	Visual/text	0.094	H5 supported
6	Positive comment sentiment	Textual interaction	0.087	H6 not supported
7	Village scene salience	Visual/cultural	0.079	H7 supported
8	Narrative clarity	Textual semantic	0.072	Positive
9	Hashtag relevance	Platform/text	0.061	Positive
10	Dialect marker density	Text/audio	0.058	Positive when explained
11	Audio rhythm density	Audio	0.054	Nonlinear
12	Instrument visibility	Visual/audio	0.049	Positive
13	Video duration	Metadata	0.046	Inverted U-shaped
14	Creator follower count	Metadata	0.041	Weak positive
15	Upload time slot	Metadata	0.034	Platform-dependent

Figure 5 shows the magnitude and direction of feature contributions to model output. Early engagement, cultural embeddedness, and youth-oriented interpretive support display the widest SHAP distributions and therefore the largest predictive contributions. Higher and lower feature values are shown in relation to their conditional contribution to the model output rather than as causal effects.

Table 10 provides the numerical ranking corresponding to Figure 5. The ranking indicates that 24-h early engagement was the strongest predictor, while cultural embeddedness and youth-oriented interpretive support ranked immediately after it. This pattern shows that the model did not rely solely on early traffic signals; culturally meaningful and interpretively accessible content features also made substantial predictive contributions.

The most influential feature was the 24-h early engagement score, which is consistent with the association between early audience response and later dissemination. However, cultural embeddedness and youth-oriented interpretive support ranked second and third, indicating that the model did not rely solely on early traffic signals. Videos that presented recognizable inheritors, local scenes, dialect singing, traditional instruments, and contextual explanations tended to receive higher model outputs for 30-day dissemination effectiveness under the observed data distribution.

The association of dialect marker density with model output was conditional. Dialectal content had a positive predictive contribution when accompanied by subtitles or explanation, but its contribution weakened when no interpretive support was provided. This result is important for blind ballad artists' narrative singing traditions because dialect and local expressions are core cultural features, but they may also create comprehension barriers for young audiences outside the community. Audio rhythm density showed a nonlinear model relationship. Moderate rhythm density was associated with higher model outputs, whereas extremely slow or highly fragmented audio segments tended to receive lower contributions. These SHAP patterns should not be interpreted as intervention effects. For example, a positive SHAP value for subtitle support indicates that subtitles contributed positively to the model output conditional on the observed feature distribution and correlations; it does not establish that experimentally adding subtitles would necessarily cause dissemination to increase.

### 5.6 Structural equation modeling results

The measurement model showed acceptable reliability and validity. Table 11 reports the individual outer loadings and construct-level reliability and convergent-validity statistics. All outer loadings exceeded 0.70. Cronbach's α ranged from 0.816 to 0.892, composite reliability from 0.846 to 0.909, and AVE from 0.579 to 0.713.

**Table 11:** Measurement model reliability and convergent validity.

Construct	Indicator loadings	Cronbach's α	Composite reliability	AVE
Digital exposure (DE)	0.724, 0.751, 0.777, 0.789	0.816	0.846	0.579
Emotional resonance (ER)	0.758, 0.786, 0.811, 0.833	0.842	0.875	0.636
Cultural identity (CI)	0.801, 0.824, 0.846, 0.861	0.871	0.901	0.694
Learning participation (LP)	0.786, 0.807, 0.836, 0.842	0.853	0.890	0.669
Transmission intention (TI)	0.806, 0.834, 0.855, 0.881	0.892	0.909	0.713

Discriminant validity is reported in Table 12. The square root of AVE for each construct exceeded its correlations with the remaining constructs, and all HTMT ratios were below 0.85. SRMR was 0.061. Full collinearity VIF values ranged from 1.214 to 2.306. Together with the Harman single-factor result of 31.8%, these results did not indicate that measurement overlap or common method variance dominated the respondent-level model.

Table 12Discriminant validity.

**Table:** 

Panel A. Fornell–Larcker criterion					
Construct	DE	ER	CI	LP	TI
**DE**	0.761				
**ER**	0.526	0.797			
**CI**	0.365	0.515	0.833		
**LP**	0.303	0.386	0.484	0.818	
**TI**	0.315	0.342	0.425	0.518	0.844

**Table:** 

Panel B. HTMT ratios	
Construct pair	HTMT
DE–ER	0.642
DE–CI	0.451
DE–LP	0.389
DE–TI	0.403
ER–CI	0.628
ER–LP	0.473
ER–TI	0.441
CI–LP	0.594
CI–TI	0.523
LP–TI	0.637

Bold values on the diagonal in Panel A represent the square root of the average variance extracted (AVE) for each construct.

Table 13 reports the structural path results. Digital exposure was positively associated with emotional resonance. Emotional resonance was positively associated with cultural identity. Cultural identity was positively associated with learning participation. Learning participation showed the strongest direct association with transmission intention. Digital exposure had a weaker positive direct association with transmission intention, whereas the ER → TI direct path did not reach the conventional significance threshold.

**Table 13:** Structural equation modeling path results.

Path	Standardized coefficient	*t*-value	*p*-value	Result
Digital exposure → Emotional resonance	0.526	11.96	< 0.001	Supported
Emotional resonance → Cultural identity	0.434	8.88	< 0.001	Supported
Cultural identity → Learning participation	0.397	7.78	< 0.001	Supported
Learning participation → Transmission intention	0.461	9.43	< 0.001	Supported
Digital exposure → Transmission intention	0.116	2.12	0.034	Supported
Emotional resonance → Transmission intention	0.089	1.84	0.066	Not supported
Cultural identity → Transmission intention	0.218	4.21	< 0.001	Supported

Figure 6 displays the theory-specified structural model and standardized coefficients. The observed coefficient pattern is statistically consistent with the hypothesized sequence from exposure through emotional resonance, cultural identity, and learning participation, but the cross-sectional design does not establish that these constructs occur in this temporal order or exclude reciprocal relationships.

**Figure 6:**
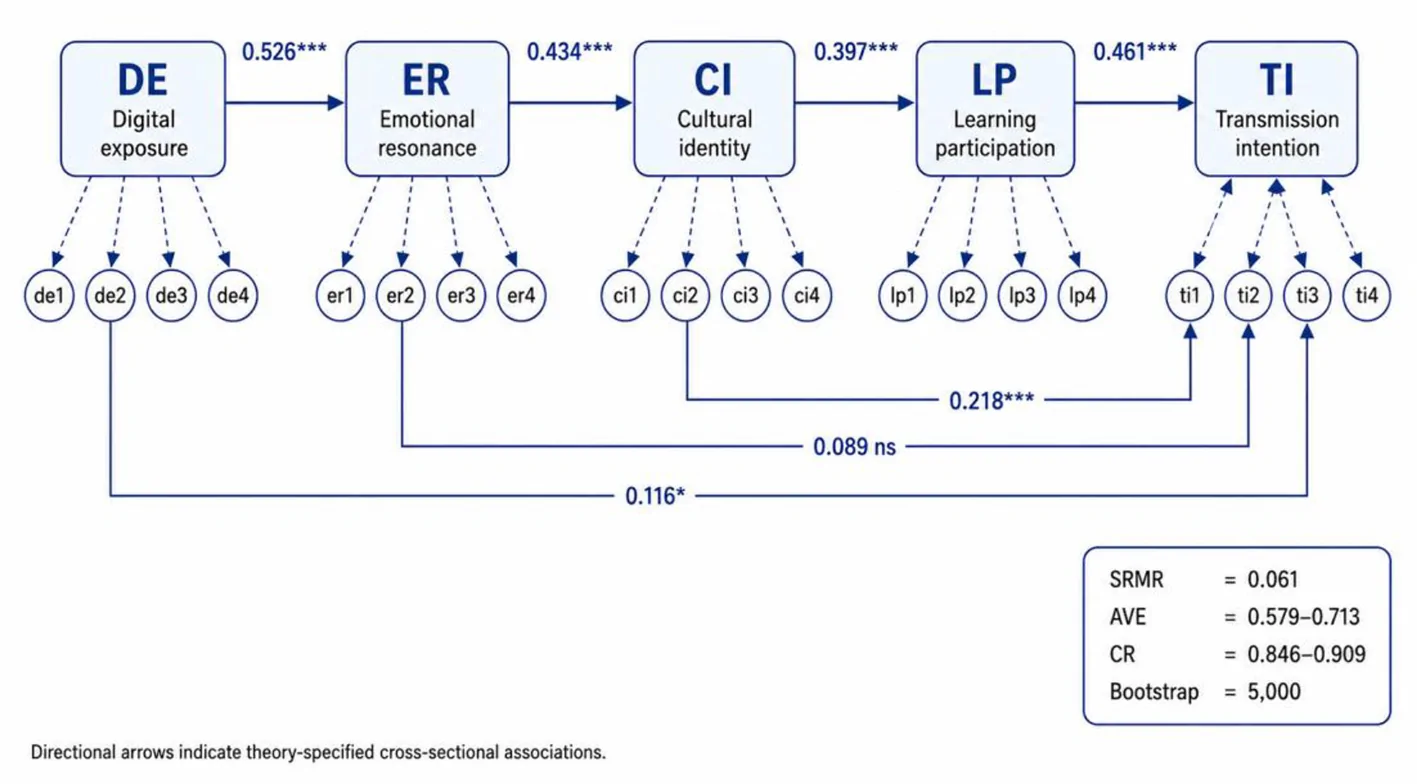
Structural equation model for intergenerational transmission intention.

Table 14 reports the indirect effects. The sequential indirect association from digital exposure to transmission intention through emotional resonance, cultural identity, and learning participation was statistically significant, supporting H8.

**Table 14:** Indirect effects in the transmission model.

Indirect path	Standardized effect	95% CI	*p*-value
DE → ER → CI	0.228	[0.171, 0.294]	< 0.001
ER → CI → LP	0.172	[0.121, 0.231]	< 0.001
CI → LP → TI	0.183	[0.129, 0.248]	< 0.001
DE → ER → CI → LP → TI	0.042	[0.025, 0.067]	< 0.001

The PLS-SEM results are consistent with the proposition that digital exposure alone provides an incomplete account of transmission intention. The observed indirect association passes statistically through emotional resonance, cultural identity, and learning participation, while learning participation has the largest direct coefficient for transmission intention. These relationships are interpreted as cross-sectional associations rather than evidence that one construct temporally causes the next.

### 5.7 Heterogeneity analysis and field interpretation

Respondent-level subgroup analysis indicated stronger transmission-related associative patterns among participants aged 16–25 than among those aged 26–35 and among village-origin youth compared with urban-only youth. These differences are interpreted cautiously because the questionnaire was not designed as a probability sample for subgroup population inference. One possible interpretation is that prior familiarity with short-video communication or local cultural contexts is associated with how subtitles, dialect, performer narratives, and village scenes are processed. Detailed subgroup results are provided in Supplementary Table S7.

The field interviews provided interpretive support for the quantitative results. Blind artists and local cultural workers emphasized that digital media expanded the visibility of performances beyond village settings, but they also expressed concern that highly fragmented clips might detach songs from their ritual, historical, or ethical context. Several young respondents reported that they first encountered blind ballad artists through online clips but developed deeper interest only after viewing subtitles, lyric explanations, or stories about the artists' life experiences.

Figure 7 presents anonymized or consent-authorized examples of performance settings, intergenerational learning, digital interpretive interfaces, and the principal qualitative themes.

**Figure 7:**
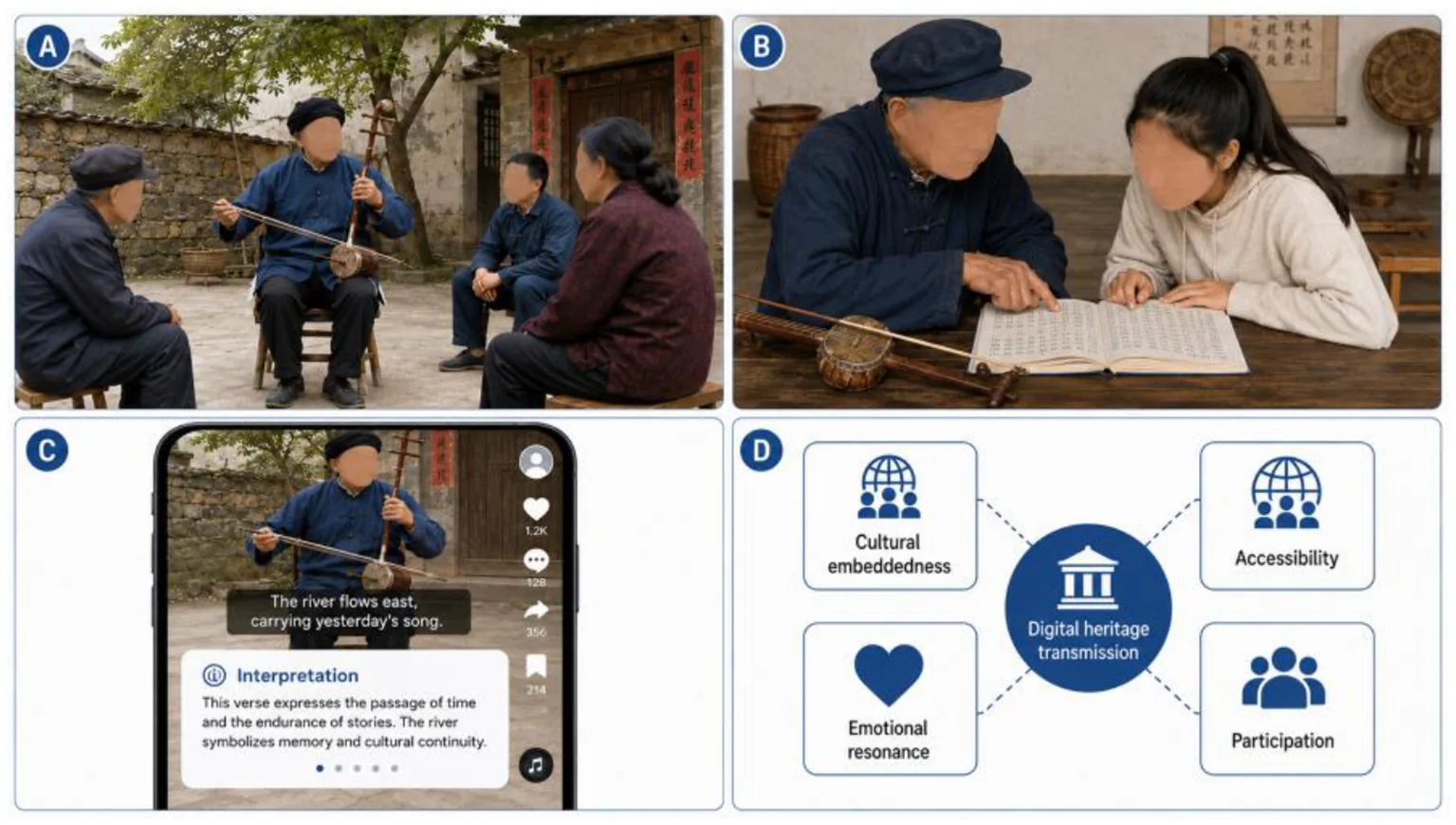
Fieldwork and digital representation examples.

Interview coding showed four recurring themes: cultural embeddedness, accessibility, emotional resonance, and participation. Cultural embeddedness was associated with real inheritors, local scenes, dialectal singing, and traditional instruments. Accessibility was associated with subtitles, explanations, and youth-oriented editing. Emotional resonance emerged when viewers connected the artist's performance with local memory, family stories, or rural life. Participation appeared when young people expressed willingness to share videos, attend local performances, learn basic singing segments, or record family elders' memories.

These qualitative themes are consistent with the predictive and respondent-level findings while providing contextual interpretations that cannot be inferred from platform statistics alone. They do not constitute independent causal verification of the quantitative associations.

## 6 Discussion

### 6.1 Cultural embeddedness as a measurable dissemination factor

The first contribution of this study is the construction of a heritage-oriented multimodal feature system. Existing digital ICH studies often focus on platform visibility, user adoption, or digital experience. This study shows that culturally meaningful features, including cultural embeddedness, inheritor visibility, village scene salience, dialect markers, and traditional instruments, are important predictors of dissemination effectiveness. This finding is particularly relevant to regional musical ICH because music heritage is not only an audio object but also a situated cultural practice involving performers, places, voices, narratives, and communities.

The results indicate that digital dissemination of musical ICH is associated with the joint presence of cultural embeddedness and communicative accessibility. Embedded features, such as inheritor visibility, dialectal singing, local scenes, and traditional instruments, provide cultural-context information. Interpretive features, such as subtitles, narrative explanation, and youth-oriented framing, are associated with lower comprehension barriers. The combination of these dimensions is consistent with stronger model-generated dissemination scores.

### 6.2 Interpretive support as a bridge between cultural embeddedness and youth engagement

Youth-oriented interpretive support emerged as one of the strongest predictors in both feature importance and ablation analysis. This finding does not imply that heritage content should be simplified or detached from its traditional form. Rather, it indicates that explanation, subtitles, contextual narration, and story-based editing are positively associated with dissemination outcomes for culturally specific content that may otherwise be difficult for younger audiences to interpret. For blind ballad artists' narrative singing traditions, dialect, local idioms, and moral storytelling are central cultural resources, but they may also create comprehension barriers for audiences without local knowledge. Interpretive support can therefore be understood as a communicative bridge between cultural embeddedness and youth engagement.

This result extends digital ICH research by suggesting that accessibility and cultural embeddedness need not be treated as opposing values. In digital environments, local performance contexts, visible inheritors, local dialect, and traditional instruments can coexist with interpretive devices that make their meanings more accessible.

### 6.3 Explainable AI as a bridge between prediction and cultural interpretation

The second contribution is the use of explainable AI to connect prediction and interpretation. The proposed framework achieved better performance than linear, tree-based, neural, and single-modality baselines under creator-level splitting. The improvement was moderate but stable. The framework's value lies not only in predictive performance but also in its ability to identify interpretable feature contributions associated with its outputs.

SHAP results revealed that early engagement, cultural embeddedness, youth-oriented interpretive support, inheritor visibility, subtitle support, and positive comment sentiment were important predictors. These predictive patterns can inform hypotheses for future content-design studies, but they should not be treated as causal prescriptions. For example, the present analysis cannot establish that experimentally adding subtitles or village scenes would necessarily increase dissemination. Randomized or quasi-experimental content interventions would be required to identify such effects. For cultural institutions, the findings suggest that digital preservation should not focus only on archiving recordings but may also benefit from explanatory and participatory layers around them.

AI tools should not replace field-based cultural interpretation. Rather, they should help identify patterns that are then interpreted through local knowledge, community perspectives, and heritage-specific criteria. This study follows this principle by combining multimodal feature extraction with expert coding and field interviews.

### 6.4 From engagement prediction to transmission-oriented digital heritage design

The third contribution is the integration of online dissemination and field-based transmission evidence. The structural equation model identified a significant statistical indirect association between digital exposure and transmission intention through emotional resonance, cultural identity, and learning participation. The observed pattern is consistent with, but does not establish, a sequence in which exposure is related to affective engagement, identity relevance, learning-oriented participation, and transmission intention. This distinction challenges a traffic-oriented understanding of digital heritage because platform attention alone is not equivalent to sustained transmission.

For blind ballad artists' narrative singing traditions, this distinction is especially important. These traditions historically depend on face-to-face listening, local memory, and community recognition. Digital media can expand reach, but online exposure should not be equated with sustained transmission. The results also highlight the intention–behavior gap. Behavioral intention is a theoretically meaningful proximal outcome because intentions represent readiness to act and are commonly associated with subsequent behavior (Ajzen, 1991). However, intention is not equivalent to behavior. Meta-analytic evidence shows that even substantial changes in intention translate only partially into observable behavioral change (Webb and Sheeran, 2006). In the present context, a young viewer may intend to learn a narrative-singing segment, attend a performance, or record an elder's oral history but may still lack access to an inheritor, a community learning space, repertoire materials, transportation, time, peer support, or institutional opportunities. Digital emotional resonance and identity relevance may therefore be associated with motivational readiness, whereas actual intergenerational participation additionally depends on offline community infrastructure. Accordingly, transmission-oriented digital heritage design should distinguish online psychological engagement from the social and institutional conditions required for sustained cultural practice. These implications are also consistent with recent work on augmented reality-mediated heritage experience and digital sustainability of heritage dissemination (Chatsiopoulou et al., 2025; Yan et al., 2025a).

### 6.5 Practical implications

The findings provide several practical implications. First, digital videos of regional musical ICH may benefit from retaining visible inheritors, local scenes, traditional instruments, and dialectal elements. Second, subtitles and concise explanations can be used to improve interpretive accessibility, especially for young and geographically distant audiences. Third, platforms and cultural institutions should support metadata systems that link videos to artists, repertoires, locations, and transmission histories. Fourth, youth participation should be encouraged through learning-oriented clips, school workshops, community recording projects, and intergenerational storytelling activities. Fifth, AI tools should be developed with community participation to avoid reducing living heritage to decontextualized media fragments. These recommendations are based on observational predictive and associative evidence and should be tested through future intervention or longitudinal research before being interpreted as demonstrated causal strategies.

### 6.6 Limitations

This study has limitations. First, the dataset was collected from three major Chinese platforms. Although these platforms are relevant to youth-oriented cultural dissemination, the findings may not fully generalize to other media environments. Platform ecology represents a more specific external-validity limitation. Bilibili, Douyin, and Kuaishou differ in audience composition, recommendation practices, creator cultures, interaction affordances, and the social meaning attached to heritage-oriented content. These characteristics may not correspond directly to YouTube Shorts, Instagram Reels, or other global short-video environments, where social-network structures, creator incentives, moderation practices, linguistic diversity, and commenting or sharing conventions may differ. The relative predictive importance of early engagement, subtitles, village scenes, or creator-related cues should therefore not be assumed to transfer unchanged across platforms. Cross-platform replication using harmonized feature definitions and platform-specific calibration is required before international generalization. Second, audio and visual quality varied across user-generated videos. Background noise, compression, and fragmented editing may have affected audio and visual feature extraction. Only conservative audio preprocessing was used, and no aggressive denoising or neural source separation was applied. This preserved dialectal and musical information but left some recording-quality variation unresolved. Future heritage-oriented audio architectures should investigate noise-robust representation learning, recording-quality indicators, and quality-aware multimodal weighting while preserving culturally meaningful vocal and instrumental characteristics. Third, although the study used field interviews and questionnaires, the transmission mechanism was measured mainly through intention rather than long-term observed behavior. This limitation should be interpreted in light of the intention–behavior gap. Transmission intention is a useful proximal psychological indicator, but actual participation requires opportunities and resources outside the digital environment. Longitudinal follow-up should therefore measure behaviors such as attending performances, repeated learning, apprenticeship contact, repertoire practice, community volunteering, or oral-history recording. Fourth, AI-based feature extraction may still miss subtle cultural meanings that require local knowledge. Fifth, although the structural equation model revealed significant associations among digital exposure, emotional resonance, cultural identity, learning participation, and transmission intention, the cross-sectional survey design does not allow strong causal claims. The directional structural model cannot establish temporal precedence, eliminate reverse causality, or rule out omitted common antecedents. Longitudinal panel data, cross-lagged designs, quasi-experiments, and experimentally manipulated digital content would be required to test temporal and causal propositions. Sixth, the questionnaire deliberately included only respondents with prior exposure to relevant digital content or local heritage activities. This exposure-conditioned purposive sample was appropriate for examining psychological variation among people capable of evaluating the target content, but it limits generalization to the broader youth population, including individuals with no prior awareness of the tradition.

## 7 Conclusion

This study developed an AI-assisted multimodal analytical framework for examining digital-media-enabled dissemination and intergenerational transmission of regional musical intangible cultural heritage. Taking blind ballad artists' narrative singing traditions in Jindongnan and the Taihang Mountains as the empirical case, the study integrated online videos, 24-h early engagement records, textual content, audio tracks, visual frames, questionnaires, and field interviews.

The proposed framework predicted 30-day dissemination effectiveness under a leakage-controlled setting and achieved stable performance across creator-level splits. Explainability analysis showed that model outputs were associated not only with early engagement but also with cultural embeddedness, youth-oriented interpretive support, inheritor visibility, subtitle support, village scene salience, and positive comment sentiment. Structural equation modeling further identified significant statistical associations linking digital exposure, emotional resonance, cultural identity, learning participation, and transmission intention, including a significant sequential indirect association. Because these results are based on cross-sectional respondent data, they do not establish temporal or causal ordering.

The combined findings suggest that transmission-oriented digital engagement is associated with culturally embedded representation, accessible interpretation, and learning-oriented participation, while actual intergenerational transmission remains dependent on offline opportunities and community infrastructure. Future research should incorporate longitudinal tracking, expand to more musical heritage forms, and develop community-centered AI tools that allow inheritors, researchers, and young learners to jointly annotate, interpret, and transmit living musical heritage. In particular, longitudinal panel studies, behavioral follow-up, cross-platform replication, and quasi-experimental or experimental content designs are needed to determine temporal ordering and to test whether changes in digital exposure and interpretive design are followed by sustained learning and observable participation in heritage transmission.

## Data Availability

The original contributions presented in the study are included in the article/Supplementary material, further inquiries can be directed to the corresponding author.
