# Supplementary material for: AI-assisted multimodal analysis of digital media-enabled intergenerational transmission of regional musical intangible cultural heritage: evidence from blind ballad artists' narrative singing in Jindongnan and the Taihang mountains
Source: Front Psychol. 2026 Sep 29;17:1953191. doi: 10.3389/fpsyg.2026.1953191 (PMC13658506; doi:10.3389/fpsyg.2026.1953191)
Supplement: Supplementary file 1 [file Supplementary_File_1.docx]

**Supplementary Material**

# Supplementary Tables

**Supplementary Table S1. Comparison between retained and excluded videos.**

| **Variable** | **Retained videos** | **Excluded videos** | **Standardized difference** |
| --- | --- | --- | --- |
| Creator followers, log | 6.42 | 6.37 | 0.04 |
| Video length, seconds | 148.3 | 151.7 | 0.03 |
| Cultural authenticity/embeddedness | 0.438 | 0.426 | 0.06 |
| Subtitle support | 54.7% | 52.9% | 0.04 |

**Supplementary Table S2. Descriptive statistics of key variables.**

| **Variable** | **Mean** | **SD** | **Min** | **Max** | **Skewness** |
| --- | --- | --- | --- | --- | --- |
| 30-day dissemination effectiveness | -2.914 | 0.862 | -5.832 | 0.764 | 0.721 |
| 24-hour early engagement score | -3.187 | 0.934 | -6.104 | 0.315 | 0.804 |
| Cultural authenticity/embeddedness | 0.438 | 0.214 | 0.000 | 1.000 | 0.317 |
| Youth-oriented interpretive support | 0.362 | 0.226 | 0.000 | 1.000 | 0.544 |
| Inheritor visibility | 0.413 | 0.492 | 0.000 | 1.000 | 0.353 |
| Village scene salience | 0.368 | 0.482 | 0.000 | 1.000 | 0.548 |
| Subtitle support | 0.547 | 0.498 | 0.000 | 1.000 | -0.189 |
| Audio rhythm density | 0.516 | 0.177 | 0.083 | 0.941 | 0.102 |
| Positive comment sentiment | 0.574 | 0.193 | 0.086 | 0.982 | -0.214 |

**Supplementary Table S3. Robustness checks.**

| **Setting** | **MAE ↓** | **RMSE ↓** | **R² ↑** | **Interpretation** |
| --- | --- | --- | --- | --- |
| Creator-level split | 0.412 ± 0.014 | 0.552 ± 0.018 | 0.706 ± 0.020 | Main setting; prevents creator leakage |
| Random split | 0.389 ± 0.012 | 0.526 ± 0.016 | 0.735 ± 0.018 | Higher performance due to creator similarity |
| $\lambda=0.25$ | 0.416 ± 0.015 | 0.558 ± 0.019 | 0.699 ± 0.021 | Stable under lower breadth weight |
| $\lambda=0.35$ | 0.412 ± 0.014 | 0.552 ± 0.018 | 0.706 ± 0.020 | Main setting |
| $\lambda=0.45$ | 0.420 ± 0.016 | 0.563 ± 0.020 | 0.694 ± 0.023 | Slightly weaker when breadth is overweighted |
| Expert-weighted embeddedness | 0.414 ± 0.015 | 0.555 ± 0.019 | 0.703 ± 0.021 | Similar to equal-weight score |

**Supplementary Table S4. Measurement model quality and model fit.**

| **Indicator** | **Recommended threshold** | **Result** |
| --- | --- | --- |
| Outer loadings | >0.70 preferred | 0.708–0.873 |
| Cronbach’s alpha | >0.70 | 0.816–0.892 |
| Composite reliability | >0.70 | 0.846–0.909 |
| AVE | >0.50 | 0.579–0.713 |
| HTMT | <0.85 | 0.421–0.782 |
| Inner VIF | <3.3 | 1.214–2.306 |
| SRMR | <0.08 | 0.056 |
| Harman’s single-factor variance | <50% | 31.8% |
| Bootstrap samples | — | 5000 |

**Supplementary Table S5. Reliability and convergent validity of latent constructs.**

| **Construct** | **Items** | **Cronbach’s alpha** | **Composite reliability** | **AVE** |
| --- | --- | --- | --- | --- |
| Digital exposure | 5 | 0.848 | 0.883 | 0.602 |
| Emotional resonance | 5 | 0.874 | 0.904 | 0.654 |
| Cultural identity | 4 | 0.892 | 0.909 | 0.713 |
| Learning participation | 4 | 0.816 | 0.846 | 0.579 |
| Transmission intention | 5 | 0.867 | 0.896 | 0.634 |

**Supplementary Table S6. Latent constructs and representative questionnaire items.**

| **Construct** | **Abbreviation** | **Example item** |
| --- | --- | --- |
| Digital exposure | DE | I frequently encounter related narrative singing content through digital media. |
| Emotional resonance | ER | These videos make me feel emotionally connected with local cultural memory. |
| Cultural identity | CI | These performances strengthen my recognition of regional cultural heritage. |
| Learning participation | LP | I am willing to learn more about the repertoire, lyrics, or performance form. |
| Transmission intention | TI | I am willing to share, support, or participate in the transmission of this heritage. |

**Supplementary Table S7. Heterogeneity analysis.**

| **Group** | **MAE ↓** | **R² ↑** | **Strongest mechanism** | **Interpretation** |
| --- | --- | --- | --- | --- |
| Age 16–25 | 0.399 | 0.721 | Digital exposure → Emotional resonance | Younger users respond strongly to short explanatory videos |
| Age 26–35 | 0.431 | 0.674 | Cultural identity → Learning participation | Older youth rely more on identity-based interpretation |
| Village-origin youth | 0.386 | 0.739 | Cultural identity → Transmission intention | Prior familiarity strengthens cultural interpretation |
| Urban-only youth | 0.446 | 0.652 | Youth-oriented interpretive support → Emotional resonance | Explanation reduces cultural distance |
| Bilibili videos | 0.429 | 0.681 | Subtitle and narrative clarity | Longer comments support interpretation but increase variance |
| Short-video platforms | 0.405 | 0.715 | Visual scene and rhythm density | Short clips benefit from immediate audiovisual cues |
